# Gut Microbiota-Mediated Transformation of Coptisine Into a Novel Metabolite 8-Oxocoptisine: Insight Into Its Superior Anti-Colitis Effect

**DOI:** 10.3389/fphar.2021.639020

**Published:** 2021-03-30

**Authors:** Gaoxiang Ai, Ziwei Huang, Juanjuan Cheng, Jianhui Xie, Huifang Zeng, Yuhong Liu, Yucui Li, Xiaoqi Huang, Jiannan Chen, Ziren Su

**Affiliations:** ^1^ School of Pharmaceutical Sciences, Guangzhou University of Chinese Medicine, Guangzhou, China; ^2^ The First Affiliated Hospital of Chinese Medicine, Guangzhou University of Chinese Medicine, Guangzhou, China; ^3^ State Key Laboratory of Dampness Syndrome of Chinese Medicine, The Second Affiliated Hospital of Guangzhou University of Chinese Medicine, Guangzhou, China; ^4^ Guangdong Provincial Key Laboratory of Clinical Research on Traditional Chinese Medicine Syndrome, The Second Affiliated Hospital of Guangzhou University of Chinese Medicine, Guangzhou, China

**Keywords:** 8-oxocoptisine, gut microflora, metabolite, ulcerative colitis, NF-κB, NLRP3 inflammasome

## Abstract

Coptisine (COP) is a bioactive isoquinoline alkaloid derived from *Coptis Chinemsis* Franch, which is traditionally applied for the management of colitis. However, the blood concentration of COP was extremely low, and its gut microbiota-mediated metabolites were thought to contribute to its prominent bioactivities. To comparatively elucidate the protective effect and underlying mechanism of COP and its novel gut microbiota metabolite (8-oxocoptisine, OCOP) against colitis, we used dextran sulfate sodium (DSS) to induce colitis in mice. Clinical symptoms, microscopic alternation, immune-inflammatory parameters for colitis were estimated. The results indicated that OCOP dramatically ameliorated disease activity index (DAI), the shortening of colon length and colonic histopathological deteriorations. OCOP treatment also suppressed the mRNA expression and release of inflammatory mediators (TGF-β, TNF-α, IL-6, IL-18, IL-1β and IFN-γ) and elevated the transcriptional and translational levels of anti-inflammatory cytokine (IL-10) as well as the mRNA expression levels of adhesion molecules (*ICAM-1* and *VCAM-1*). Besides, the activation of NF-κB pathway and NLRP3 inflammasome was markedly inhibited by OCOP. Furthermore, OCOP displayed superior anti-colitis effect to COP, and was similar to MSZ with much smaller dosage. Taken together, the protective effect of OCOP against DSS-induced colitis might be intimately related to inhibition of NF-κB pathway and NLRP3 inflammasome. And the findings indicated that OCOP might have greater potential than COP to be further exploited as a promising candidate in the treatment of colitis.

**GRAPHICAL ABSTRACT:**
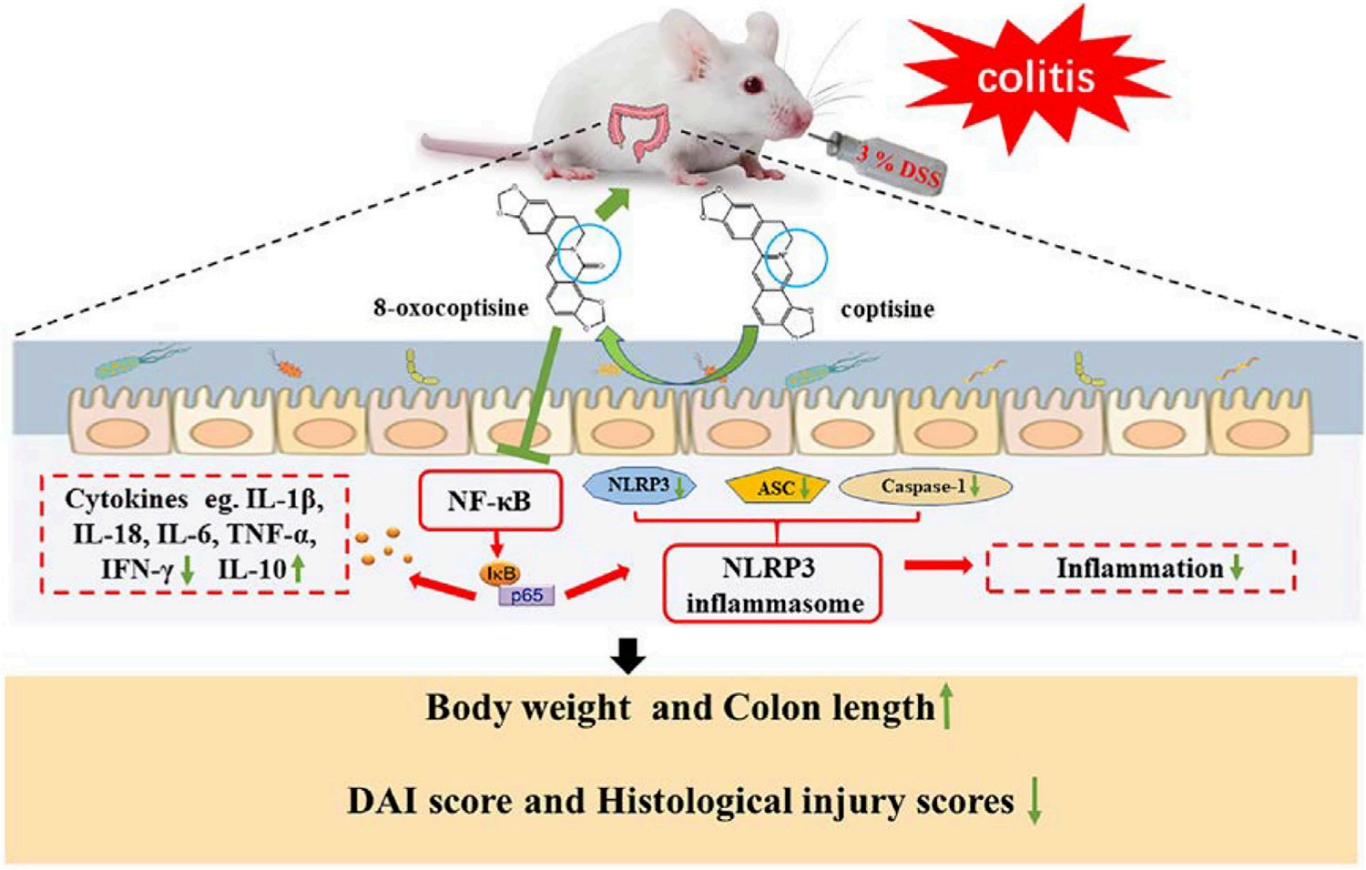


## Introduction

Ulcerative colitis (UC), a major form of inflammatory bowel disease (IBD), is an idiopathic long-term inflammatory disorder of the colon and rectum characterized by abdominal pain, fatigue, rectal bleeding, persistent diarrhea and abdominal cramps (Feuerstein and Cheifetz, 2014). UC is more prevalent in developed countries, and results in considerable economic burden for medical systems, unsatisfactory life quality and even life-threatening (Ananthakrishnan, 2015; Luo et al., 2020).

For medical management of UC, current therapeutic agents mainly focus on nonsteroidal anti-inflammatory agents, corticosteroids and immunosuppressants, which can be effective in the early stage of disease. However, these pharmaceutical therapies would be compromised due to incomplete efficacy and frequent side effects (Kellermayer, 2017; Ahmad and Kumar, 2018). Hence, it is of great importance to explore the candidate compounds for UC treatment.

Traditional Chinese medicine has been used as a fundamental therapeutic arsenal for diseases with enduring clinical practice and reliable therapeutic efficacy in China (Shaker et al., 2014). Rhizoma Coptidis (the dried rhizome of *Coptisine Chinemsis* Franch), which is officially recorded in the Chinese pharmacopoeia (known as Huanglian in Chinese), is widely applied for various diseases including dysentery (also known as UC) (Wu et al., 2014; Morita et al., 2016; Dong et al., 2019). Coptisine (COP), the characteristic active constituent of Rhizoma Coptidis with typical natural benzyltherahydroisoquinoline-type alkaloids skeleton as berberine, possesses a broad spectrum of prominent bioactivities including anti-inflammatory (Zielinska et al., 2020), anti-diabetes (Shi et al., 2019), anti-malaria (Lang et al., 2018) and anti-cancer effects (Ya et al., 2020), which is a promising active component to treat UC. Nevertheless, low oral bioavailability of COP is difficult to expound its various pharmacological activities (Wu et al., 2019).

The liver, one of the body’s major organs, plays a fundamental role in drug metabolism and bioconversion. However, the AUC_0-t_ value of COP after intraportal administration was markedly higher than that by intraduodenal administration, which indicated the main metabolic organ of COP was not the liver (Liu et al., 2015; Ren et al., 2019; Lu et al., 2020). In addition, many studies have reported that COP was mainly metabolized in the intestine with only low level distributed in other tissues (kidney, spleen, liver, heart, and muscle) (Su et al., 2015; Hu et al., 2019). Therefore, it was deduced that COP was firstly metabolized by intestine in large amount. Gut microflora involved in the drug metabolism is considered the “hidden” organ in the intestine, which may lead to the production of new metabolites with altered bioactivities. Intestine microbiota can transform COP into various metabolites by decarbonization, reduction and other reactions. However, inferior pharmacological effects of these gut microbiota-mediated metabolites are difficult to explain the broad bioactivities of COP (Li et al., 2015). Hence, it is interesting to explore additional metabolic pathways to explain the prominent pharmacological activities of COP.

In the present study, COP was firstly found to be transformed into 8-oxocoptisine (OCOP), an oxidized protoberberine alkaloid with more active lactam ring, by oxidation reaction. It has been reported that OCOP exhibited appreciable anti-inflammatory, anti-tumor, anti-fungal and cardiovascular-protective effects (Hirano et al., 2001; Min et al., 2006). And in our preliminary study, COP and OCOP were both found to exhibit beneficial effects in treating UC. However, comparative study on the potential functional implication and connection of bioactive effects between COP and its metabolites is scare. Notably, in our previous work, berberine with the same core structure as COP, was found to be transformed into a novel oxidative metabolite oxyberberine (OBB) by gut microbiota-medicated oxidation reaction, which exhibited predominant anti-inflammatory (Li et al., 2020) and anti-colitis effects with more favorable safety profile (Tan et al., 2016; Li et al., 2020). Therefore, we speculated that OCOP, an important oxidative metabolite of COP, may serve as a promising bioactive candidate, which motivated us to further investigate its pharmacological activities such as anti-colitis effect.

As part of our ongoing search for anti-colitis candidates, the current research was designed to comparatively explore the potential effects of COP and its intestinal oxidative metabolite OCOP on dextran sulfate sodium (DSS)-induced mice colitis and unravel the potential mechanism. Our results indicated that OCOP eminently alleviated acute colitis elicited by DSS, which exhibited similar anti-UC effect to the reference drug mesalazine (MSZ) with much smaller dosage and superior to COP via regulation of NF-κB pathway and NLRP3 inflammasome. This is, to our knowledge, the first study to expound the anti-UC effect and potential mechanism of OCOP *in vivo*. Our study presents an intriguing case and paradigm to illustrate the significance of the gut microbiota in enhancing the therapeutic efficacy of natural products. It also offers further empirical evidence for the traditional application of Rhizome Coptidis against diarrhea and dysentery. Furthermore, it may provide novel insight into the potential usefulness of OCOP as a novel lead compound against UC.

## Materials and Methods

### Reagents and Chemicals

COP (purity >98%) was purchased from Shanghai Yuan Ye Biotechnology Co., Ltd (Shanghai, China). Dextra sulfate sodium was obtained from MP Biomedical (Irvine, CA, USA). Cefadroxil (purity >99%), terramycin (purity >98%) and erythromycin (purity >99%) were purchased from Dalian Meilun Biotechnology Co., Ltd (Dalian, China). Mesalazine (MSZ) was obtained from Losan Pharma GmbH, Germany. ELISA kits (TNF-α, IL-6, IL-10, IL-18, IL-1β, TGF-β, and IFN-γ) were obtained from Shanghai Enzyme-linked Biotechnology Co., Ltd (Shanghai, China). BCA kit and myeloperoxidase (MPO) assay kit were obtained from the Jiancheng Bioengineering Institute of Nanjing (Nanjing, Jiangsu, China). Primary antibodies against p65, IκBα, p-IκBα, NLRP3, ASC, Capase-1, Caspase-1 p10, GAPDH and Histone H3 were purchased from Affinity Biosciences (OH, USA). All other reagents and chemicals were at least of analytical grade.

### Animals

Male BALB/C mice (22–24 g) and Kunming (KM) mice (18–22 g) were obtained from the animal center of Guangzhou University of Chinese Medicine, Guangdong Province, China. The animals were acclimated for one week prior to the initiation of the experiment. Animals were raised in a 12 h day-night rhythm under the temperature of 23–25°C and the relative humidity of 40–60%, and got access to standard forage and clean water *ad libitum*. The experiment was carried out strictly in compliance with the National Institutes of Health (NIH) Guide for the Care and Use of Laboratory Animals (eighth edition), All protocols were approved by the Animal Experiment Ethics Committee of Guangzhou University of Chinese Medicine (Permit ID: 20190223002) (Mason and Matthews, 2012).

### The Bioconversion of Coptisine by Gut Microbiota

The bioconversion of COP by gut microbiota was conducted according to the regime of intestinal metabolite of berberine in our previous work with some modification (Li et al., 2020). Firstly, for incubation, the fresh feces collected in KM mice were immediately homogenized in anaerobic culture solution. After filtration, COP was added to the intestinal bacteria solution, and the final concentration of COP in the incubation system was 100 μg/ml. The mixture solution was incubated under anaerobic condition at 37°C for 24 h. Secondly, the feces were collected for detecting and identifying metabolites within 24 h after oral administration with COP. Thirdly, to validate the role of intestinal bacteria in the process of COP metabolism, pseudo-germfree model was established following the previous method to assess the metabolic bioconversion of COP *in vivo* (Feng et al., 2015). The samples were treated with acetonitrile, vortexed for 2 min, and centrifuged at 8000 g for 10 min. The supernatant was then collected for N_2_ drying. The residue was reconstituted with 200 µL acetonitrile, then vortexed for 30 s, and filtered by 0.22 μm microporous filter. Finally, a 10-μL aliquot was injected into the LCMS/MS system for analysis.

### LC-MS/MS Instrument and Operation Conditions

Detection was performed on Shimadzu ultra-performance liquid chromatograph system hyphenated with ion trap time-of-flight mass spectrometry (Shimadzu Cooperation, Japan). Chromatographic separation was conducted on a phenomenex Luna C18 (2) 100A column (250 mm × 4.6 mm, 5 μm). The mobile phase consisted of solution (containing 0.34% sodium dodecylsulfate and 0.67% potassium dihydrogen phosphate)/acetonitrile (50:50, v/v). All the data were collected in the ESI-positive ionization detection mode within the *m/z* range of 200–2000 Da.

### Synthesis and Identification of 8-Oxocoptisne

The synthesis of OCOP was performed as previously described with minor modification (Zhang et al., 2014). Briefly, potassium ferricyanide was dissolved in the solution of 5 N NaOH in deionized water and COP was added to the solution with the ratio of 5:1. The suspension solution was conducted under reflux condition to get the crude product, which was purified using silica gel column chromatography. Then, a series of analysis technologies including NMR spectroscopy and HPLC-ESI-MS were employed to analyze the synthesized sample.

### Induction of Colitis

After acclimation for 7 days, mice were randomly assigned into six groups: Control, 3% DSS, 3% DSS with MSZ (MSZ, 200 mg/kg), 3% DSS with COP (COP, 50 mg/kg) and 3% DSS with OCOP (OCOP, 50 and 100 mg/kg). Except the control group, other groups received 3% DSS (w/v) dissolved in the sterile water for seven consecutive days to establish UC model. The control group was given the same volume of 2% Tween-80 aqueous solution. MSZ, COP, and OCOP were orally administered once daily. The body weight and the presence of stool bleeding as well as diarrhea were monitored daily throughout the experiment. On the last day, all mice were euthanized, and the entire colon tissues were collected and measured. Subsequently, the entire colon was divided into several segments proportionally and stored at −80°C for subsequent biochemical analysis.

### Assessment of Disease Activity Index

Clinical manifestations like diarrhea, stool bleeding, and body weight of all animals were monitored daily throughout the experiment. The final DAI score included the score of the above symptoms. The specific score for every index followed the previous method (Table 1) (Alex et al., 2009):

**TABLE 1:** Criteria for scoring disease activity index (DAI).

Score	Body weight (compared to the original body weight)	Stool bleeding	Stool consistency
0	No change	None	Normal
1	≤5%		Soft but still formed
2	≤6∼10%	Blood traced in stool visible	Very soft
3	≤11∼20%		Half diarrhea
4	≥20%	Totally rectal bleeding	Diarrhea

### Evaluation of Histological Changes

For histological analysis, the samples were fixed in fresh 4% paraformaldehyde (pH 7.4). After routine tissue processing, the samples were embedded in paraffin. Then these well-processed specimens were sliced to 5 μm thickness sections for Hematoxylin & Eosin staining. After H&E staining, histological scoring for colitis was performed by colon pathologist in a blinded way using a validated score system based on pathological changes in colons (Zhang et al., 2018). The final score was the sum of the following mentioned index score. The specific score rules were as previously described (Table 2) (Zhang et al., 2018).

**TABLE 2:** Histological grading criteria.

Score	Epithelial loss	Crypt damage	Reduction in the number of goblets cells	Inflammatory cell infiltration
0	no loss	no damage	none	none
1	0∼5%	5∼10% damage	mild	mild
2	5∼10%	10∼20% damage	moderate	moderate
3	more than 10% loss	more than 20% damage	severe	severe

### Measurement of Myeloperoxidase Activity in Colons

Colons from mice in each group were homogenized with saline solution on ice for 3 min using a homogenizer. The well-homogenate supernatants were collected after centrifugation (10,000 g, 4°C, 20 min). The activity of MPO was determined by MPO assay kit following the manufacturer’s protocol.

### Measurement of Cytokines Concentrations in Colonic Tissues

The extracted colon specimens were homogenized in phosphate buffer saline (pH 7.4) on ice for 3 min using a homogenizer. The well-homogenate supernatants were collected after centrifugation (10,000 *g*, 4°C, 20 min). The contents of cytokines (IL-10, TNF-α, IL-6, IL-1β, IFN-γ, TGF-β and IL-18) were determined by ELISA kits following the manufacturer’s operational protocol.

### Analysis of mRNA Expression by Real-Time Quantitative PCR in the Colon Samples

Total RNAs from colon samples were extracted using Trizol reagent in accordance with the user’s manuals. And its purity of RNA was evaluated by determining the ratio of A260/A280 (between 1.8 and 2.0). Total RNAs were employed to synthesize cDNA using a reverse transcription kit (Thermo Fisher Scientific, USA) following the manufacturer’s protocol. The levels of *IL-1β, IL-10, IL-18, IL-6, TNF-α, IFN-γ, ICAM-1,* and *VCAM-1* were analyzed by quantitative real-time PCR with FastStart Universal SYBR Green Master (Rox). The sequences of the primers are listed in Table 3. The following PCR conditions were applied for PCR amplification: pre-denaturation at 95°C for 30 s, followed by 40 cycles at 95°C for 10 s and 60°C for 30 s. The relative quantification of target gene expression was analyzed using the 2^−ΔΔCt^ method and GAPDH served as an internal control.

**TABLE 3:** Primers sequences.

Gene		Gene sequence (5’ to 3’)
*GAPDH*	Forward	GCA​CAG​TCA​AGG​CCG​AGA​ATG​G
	Reverse	GGT​GGC​AGT​GAT​GGC​ATG​GAC
*ICAM-1*	Forward	AGTCGTCCGCTTCTACC
	Reverse	CCA​GCA​CCG​TGA​ATG​TGA​TCT​CC
*VCAM-1*	Forward	TGT​GCT​GCT​ATT​GGC​TGT​GAC​TC
	Reverse	GCA​GTT​GAC​AGT​GAC​AGG​TCT​CC
*TNF-α*	Forward	GCG​ACG​TGG​AAC​TGG​CAG​AAG
	Reverse	CAT​CGG​CTG​GCA​CCA​CTA​GTT​G
*IL-1β*	Forward	GCA​CTA​CAG​GCT​CCG​AGA​TGA​AC
	Reverse	AGG​CTT​GTG​CTC​TGC​TTG​TGA​G
*IL-18*	Forward	TGC​CAT​GTC​AGA​AGA​CTC​TTG​CG
	Reverse	GGT​CAC​AGC​CAG​TCC​TCT​TAC​TTC
*IL-10*	Forward	AGC​TGG​ACA​ACA​TAC​TGC​TAA​CCG
	Reverse	CTT​CAC​CTG​CTC​CAC​TGC​CTT​G
*IFN-γ*	Forward	TTA​CTG​CCA​CGG​CAC​AGT​CAT​TG
	Reverse	TCG​CCT​TGC​TGT​TGC​TGA​AGA​AG
*IL-6*	Forward	TGA​ACA​ACG​ATG​ATG​CAC​TTG​CAG
	Reverse	TAG​CCA​CTC​CTT​CTG​TGA​CTC​CAG

### Immunohistochemical Staining

The colon samples were fixed immediately in 4% buffer formalin, and embedded in paraffin. These well-processed specimens were cut into 5 μm sections. The paraffin-fixed sections were dewaxed, rehydrated and washed in 1% PBS-Tween buffer. Sequentially, theses samples were treated with 3% hydrogen peroxide, blocked with 10% goat serum and incubated with NLRP3 and p65 primary antibodies (Affinity Bioscience, OH, USA) at 4°C overnight. These sections were incubated with the corresponding goat anti-rabbit secondary antibodies labeled with horseradish peroxidase (37°C, 30 min) (Affinity Bioscience, OH, USA). After washing thoroughly, the slides were stained with 3,3′-diaminobenzidine (DAB) followed by re-dyeing with hematoxylin. Theses slices were evaluated using the confocal laser-scanning microscope (Olympus FV1000, Tokyo, Japan).

### Western Blotting Analysis

Western blotting was used to assess the protective effect of OCOP on the NF-κB pathway and NLRP3 inflammasome. The colon segments were obtained from mice and homogenized on pre-cooled PBS. Then, they were lyzed in RIPA buffer (RIPA lysis buffer: PMSF: protein phosphatase inhibitor = 100:1:1) on ice for 30 min, which were further centrifuged at 12,000 g for 15 min to obtain the supernatants. To determine the concentration of the protein, BCA protein assay kit was performed. Total proteins from colon tissues were separated by SDS-PAGE and transferred to PVDF membrane. After being blocked in non-fat milk in Tris-buffer saline-0.1% Tween-20 (TBST) for 2 h, samples were incubated with the corresponding primary antibody p65, IκBα, p-IκBα, NLRP3, ASC, Caspase-1, Caspase-1 p10, GAPDH, and Histone H3 (Affinity Bioscience, OH, USA) overnight at 4°C. Subsequently, the membranes were incubated with horseradish peroxidase-conjugated secondary antibodies. The chemiluminescence signals were detected by ECL detection reagents and analyzed by Image J software. GAPDH and Histone H3 were used as the loading controls.

### Molecular Docking

Molecular docking can provide more detailed information for investigating the interaction between ligands (COP and OCOP) and the receptors (NLRP3, Caspase-1 and NF-κB) with the aid of AutoDock software (version 4.2.6). The 3D structures of Caspase-1 (PDB ID: 1RWK), NLRP3 (PDB ID: 6NPY) and NF-κB (PDB ID: 1NFI) were downloaded from the RCSB PDB database (http://www.rcsb.org/pdb).The crystallographic coordinates of COP and OCOP were retrieved from the PubChem Compound database (http://pubchem.ncbi.nlm.nih.gov/pccompound). The grid size along the x-, y-, *z*-axes was set at 30 × 30 × 30 with 1 Å grid spacing. Lamarckian Genetic Algorithm (LGA) was used to generate the binding pose between ligands and the receptors. The lowest binding free energy conformation between ligand and receptor was chosen as the best docking pose. Finally, graphical representations were viewed by a PyMOL graphic system.

### Statistical Analysis

Data were expressed as means ± standard deviation of the means (S.D.). Statistical analysis was performed using SPSS software (version 19.0, SPSS, Chicago, IL, USA). The statistically significant difference between two groups was evaluated using student’s t-test. One-way analysis of variance (ANOVA) followed by Dunnett’s test was used to evaluate the differences when multiple groups were compared. Statistical significance was set by the *p* value below 0.05 or the *p* value below 0.01.

## Results

### Metabolizing Coptisine by Gut Microbiota *in vitro* and *in vivo*

**FIGURE 1:**
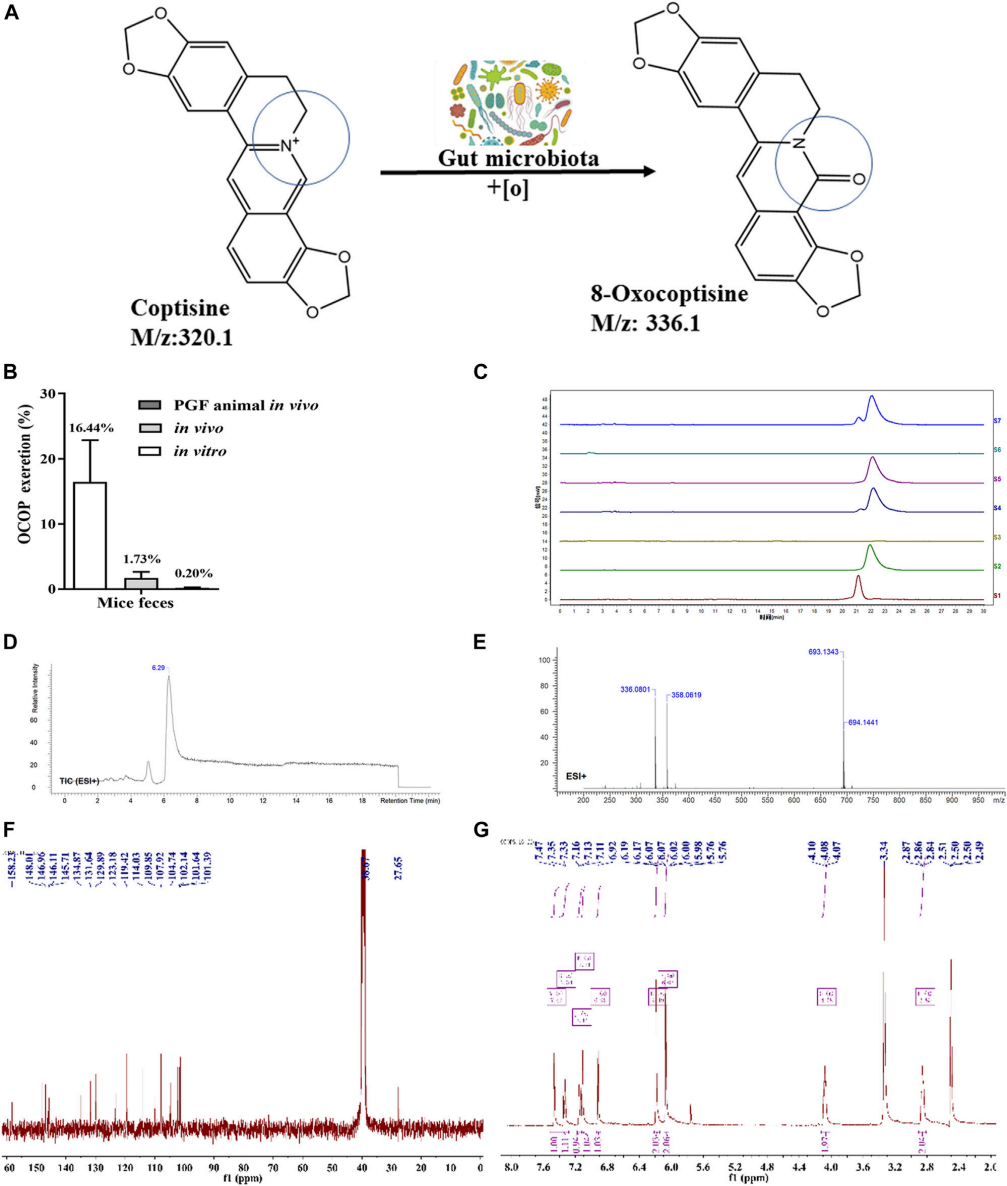
Generation of OCOP by gut microbiota **(A)** COP was metabolized into OCOP by gut microbiota **(B)** The OCOP content (%) in the mice feces after administration *in vitro* and *in vivo* for 24 h **(C)** HPLC analysis of S1 (standard solution of OCOP), S2 (standard solution of COP), S3 (the feces of normal mice), S4 (the feces of mice orally treated with 100 μg/ml COP), S5 (the feces of pseudo-germfree mice orally treated with 100 μg/ml COP), S6 (the feces of normal mice *in vitro*), and S7 (the feces of normal mice incubated with 100 μg/ml COP *in vitro*) **(D and E)** ESI^+^-MS^1^ spectrum of OCOP in methanol **(F and G)** ^1^H NMR, ^13^C-NMR spectrum data of OCOP.

As illustrated in Figure 1, HPLC analysis indicated that COP could be metabolized *in vivo*. LC-MS analysis revealed that COP was transformed into a novel metabolite OCOP (LC-ESI-MS *m/z*: 336.1) in normal and pseudo-germfree mice. However, oral treatment of mice with antibiotics could reduce OCOP generation in pseudo-germ-free mice. In normal mice, the conversion rate of COP converted to OCOP by gut microbiota *in vitro* was 16.44%. After treating with COP by oral administration, the content of OCOP was 1.73% in fresh feces of normal KM mice within 24 h. However, the production of OCOP was drastically reduced to 0.20% in pseudo-germ-free mice within 24 h. This finding suggests that COP could be metabolized to the oxidative metabolite OCOP by intestinal microbiota in normal mice.

### Synthesis and Identification of 8-Oxocoptisne

As shown in Figure 1, the mass spectrum obtained by LC-ESI-MS gave an ion peak at *m/z*: 336.1 in positive ion mode, suggesting the molecular formula of this synthetic earthy gray sandy solid was C_19_N_13_NO_5_. Its NMR spectrometry data were given below: ^1^H NMR (400 MHz, DMSO-*d*_6_) δ: 2.85 (t, *J* = 5.4Hz, 2H, NCH_2_CH_2_), 4.08 (t, *J* = 5.4Hz, 2H, NCH_2_CH_2_), 6.07 (s, 2H,OCH_2_O), 6.19 (s, 2H,OCH_2_O), 6.92 (s, 1H, ArH), 7.11 (s, 1H, ArH), 7.15 (d, *J* = 8.1Hz, 1H, ArH), 7.34 (d, *J* = 8.1Hz, 1H, ArH), 7.47 (s, 1H, -CH = ); ^13^C NMR (100 MHz, DMSO-*d*_6_) δ: 158.23, 148.01, 146.96, 146.11, 145.71, 134.87, 131.64, 129.89, 123.18, 119.42, 114.03, 109.85, 107.92, 104.74, 102.14, 101.64, 101.39, 38.67, 27.65. In summary, the synthetic earthy gray sandy solid was identified as OCOP according to the data of ^1^H- nuclear magnetic resonance (NMR) and ^13^C-NMR and mass spectrometry (Zhang et al., 2014). Meanwhile, HPLC analysis revealed that the purity of OCOP was more than 98%.

### Anti-UC Activities of 8-Oxocoptisne

#### General Clinical Symptoms of Dextran Sulfate Sodium-Induced Colitis in Mice

**FIGURE 2:**
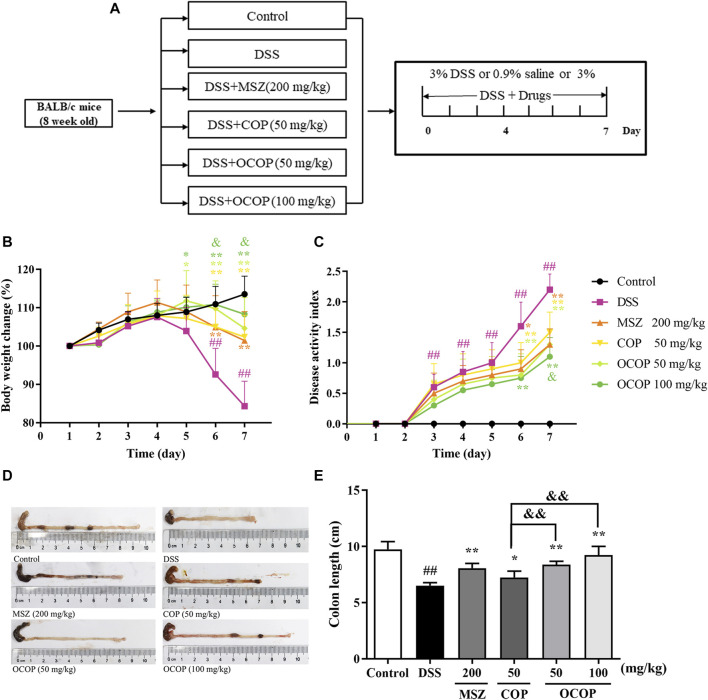
Effects of OCOP (50 and 100 mg/kg) and COP (50 mg/kg) on daily body weight changes, and disease activity score of DSS-induced colitis mice. After DSS challenge, treatments were administered once a day by intragastric gavage for a week **(A)** Schematic diagram of the experiment design **(B)** Daily body weight changes **(C)** DAI score index **(D)** Macroscopic photographs of colon lengths **(E)** The length of colon. Data are expressed as the means ± SD (n = 8–10). ^#^ *p* < 0.05, ^##^ *p* < 0.01 vs. control group; *p < 0.05, **p < 0.01 vs. DSS group; ^&^ *p* < 0.05, ^&&^ *p* < 0.01 vs. COP group.

In the present study, oral administration of DSS induced colitis in mice, which resembled human UC. Mice in the DSS group appeared rapid weight loss, stool bleeding and diarrhea and lack of viability, together with loosened hair and fecal blood. As illustrated in Figure 2, the body weight of DSS-induced mice decreased evidently during the experiment. However, compared with the DSS-induced mice model, OCOP treatment significantly improved the abovementioned pathological manifestations. Notably, OCOP (50 mg/kg) was more pharmacologically active than COP of the same dosage in ameliorating clinical symptoms of DSS-induced colitis, and exerted similar therapeutic effect to MSZ (200 mg/kg). The DAI score was consistent with the pathological manifestations.

As shown in Figure 2, in contrast to the control group, DSS-induced mice presented a progressive increase in DAI value. However, treatment with OCOP, COP and MSZ for 7 days dramatically (*p* < 0.05) lowered the DAI score of mice when compared with the model group. In addition, it is widely accepted that intestinal atrophy is positively related to the colitis (Verma et al., 2014). The colon in DSS groups was shorter than that in the control group. However, treatment with OCOP, COP and MSZ obviously (*p* < 0.05) ameliorated the shortening of colon length. It was suggested that treatment with OCOP exhibited pronounced ameliorative effect against DSS-induced experimental UC.

#### Histopathological Analysis on H&E Staining and Myeloperoxidase Activity of Dextran Sulfate Sodium-Induced Colitis in Mice

The histological architecture of the colons was observed by microscope after H&E staining. The colons from the control group showed intact surface epithelium, crypt, muscularis, mucosa, and submucosa. However, the DSS-induced group displayed excessive crypt damage with edema, collapse, or complete destruction, and a large amount of inflammatory cell infiltration. The severe inflammation contributed to higher microscopic score. However, compared with the DSS-induced group, treatment with OCOP, COP and MSZ apparently improved the degree of crypt damage. Meanwhile, less amount of inflammatory cell infiltration was observed. And the microscopic score of OCOP groups (50 and 100 mg/kg) was observably lowered in a dose-dependent manner (*p* < 0.05). Notably, OCOP-H exhibited superior therapeutic effects to COP of the same dosage and similar to MSZ.

**FIGURE 3:**
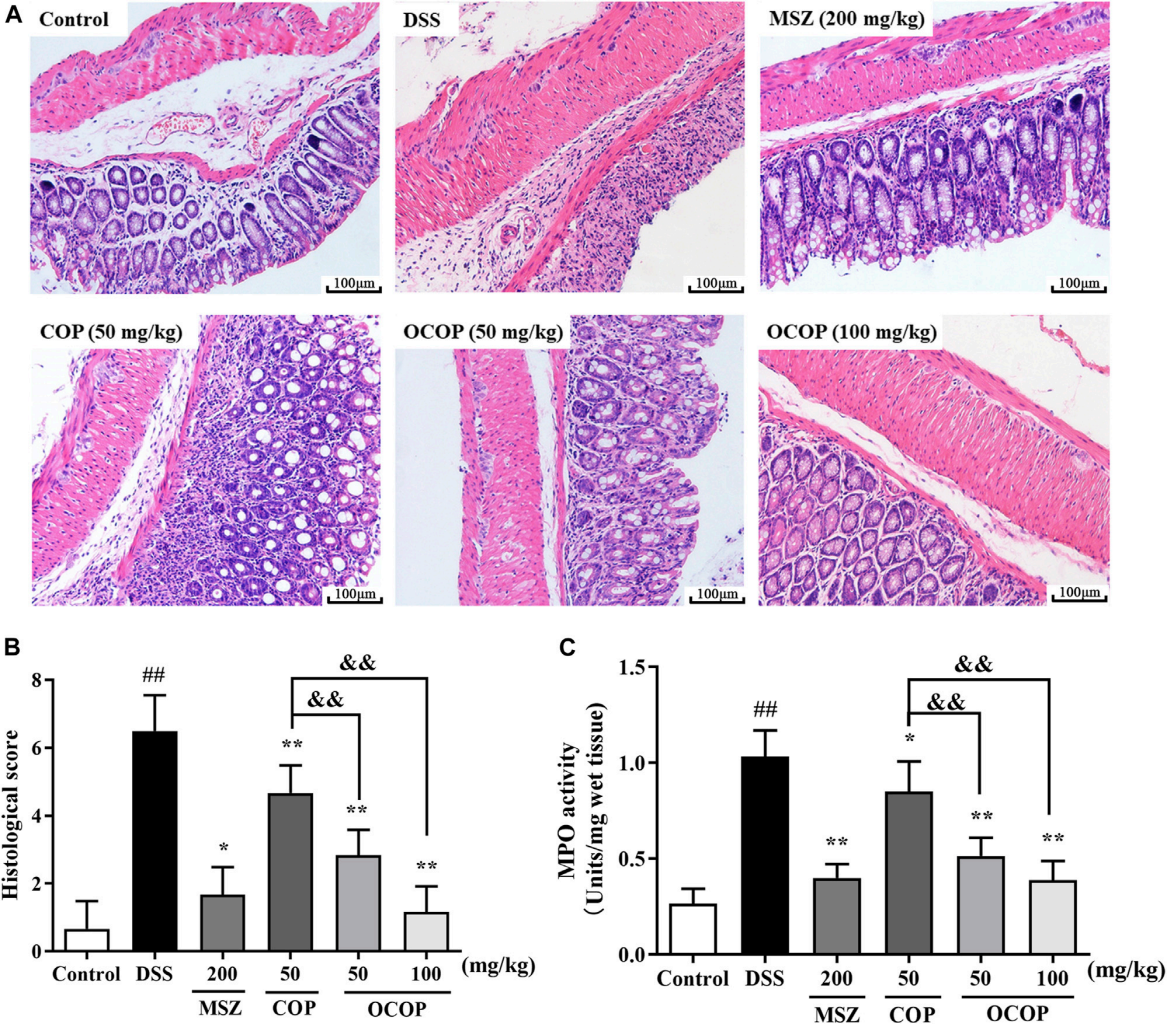
**(A)** Representative colorectal slices of H&E staining. Original magnification ×200. **(A)** control; **(B)** DSS; **(C)** MSZ; **(D)** COP (50 mg/kg); **(E)** OCOP (50 mg/kg); **(F)** OCOP (100 mg/kg) **(B)** Histological scores of each group **(C)** The MPO activity of colon tissues. Data are expressed as the means ± SD (n = 6–8). ^#^ *p* < 0.05, ^##^ *p* < 0.01 vs. control group; *p < 0.05, **p < 0.01 vs. DSS group; ^&^ *p* < 0.05, ^&&^ *p* < 0.01 vs. COP group.

MPO is a biomarker reflecting neutrophils granulocyte into the inflamed intestinal tissue. As illustrated in Figure 3, in contrast to the control group, the activity of colonic MPO was remarkedly increased in DSS group, suggesting that the colon tissues were gradually infiltrated by excessive neutrophils granulocyte. However, the elevated MPO activity was markedly (*p* < 0.05) suppressed by OCOP, COP and MSZ treatment in comparison with that of DSS group. The results suggested OCOP effectively reduced the infiltration of neutrophils granulocyte, which was superior to COP of the same dosage.

#### Effect of 8-Oxocoptisne on the Levels of Colonic Cytokines in Dextran Sulfate Sodium-Induced Mice

**FIGURE 4:**
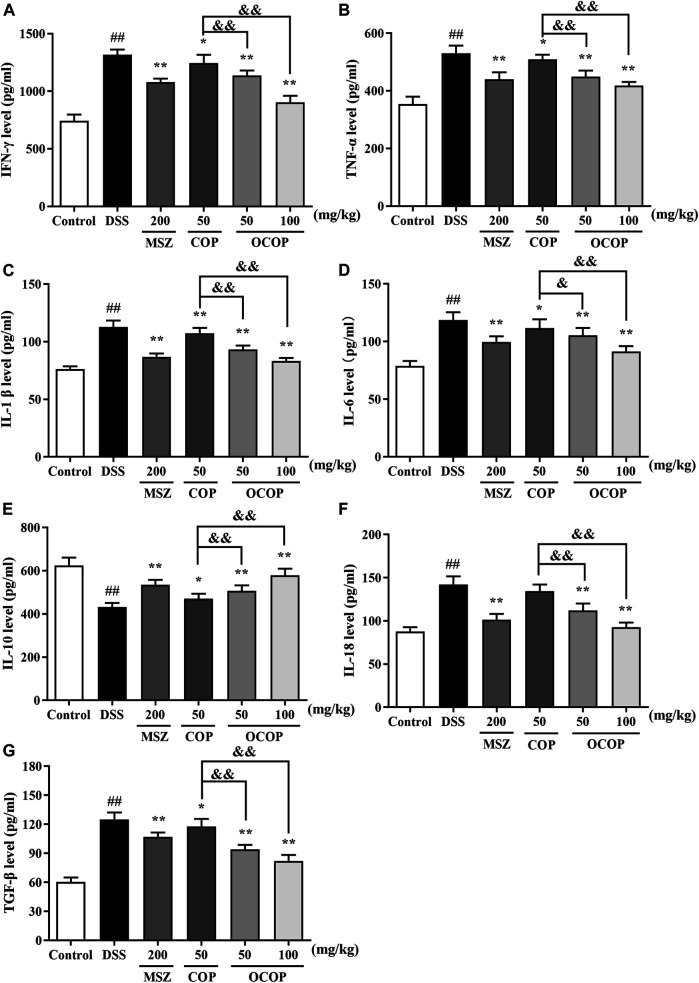
Effects of OCOP (50 and 100 mg/kg) and COP (50 mg/kg) on the productions of inflammatory cytokines IFN-γ **(A)**, TNF-α **(B)**, IL-1β **(C)**, IL-6 **(D)**, IL-10 **(E)**, IL-18 **(F)** and TGF-β **(G)** in colon tissues of DSS-induced mice. After DSS challenge, treatments were administered once a day by intragastric gavage for a week. Data are expressed as the means ± SD (n = 10). ^#^ *p* < 0.05, ^##^ *p* < 0.01 vs. control group; *p < 0.05, **p < 0.01 vs. DSS group; ^&^ *p* < 0.05, ^&&^ *p* < 0.01 vs. COP group.

As presented in Figure 4, compared with the control group, DSS treatment dramatically (*p* < 0.01) increased the levels of IL-1β, IL-6, TNF-α, IL-18, IFN-γ and TGF-β*.* In contrast, the productions of these mediators were strikingly (all *p* < 0.05) attenuated by OCOP, COP and the positive drug MSZ. On the other hand, lower level of IL-10 was observed in DSS-induced experimental colitis in parallel to the control group. However, oral administration with OCOP, COP and MSZ significantly (*p* < 0.05) elevated the level of IL-10 in comparison to the DSS group. It was noteworthy that OCOP exhibited superior effect to COP of the same dosage and similar to MSZ in modulating the inflammatory status. These results indicated that OCOP possessed favorable anti-inflammatory effect in mitigating DSS-induced colitis.

#### Effect of 8-Oxocoptisne on the mRNA Expression of Immune-Inflammation Mediators in Mice Colon

**FIGURE 5:**
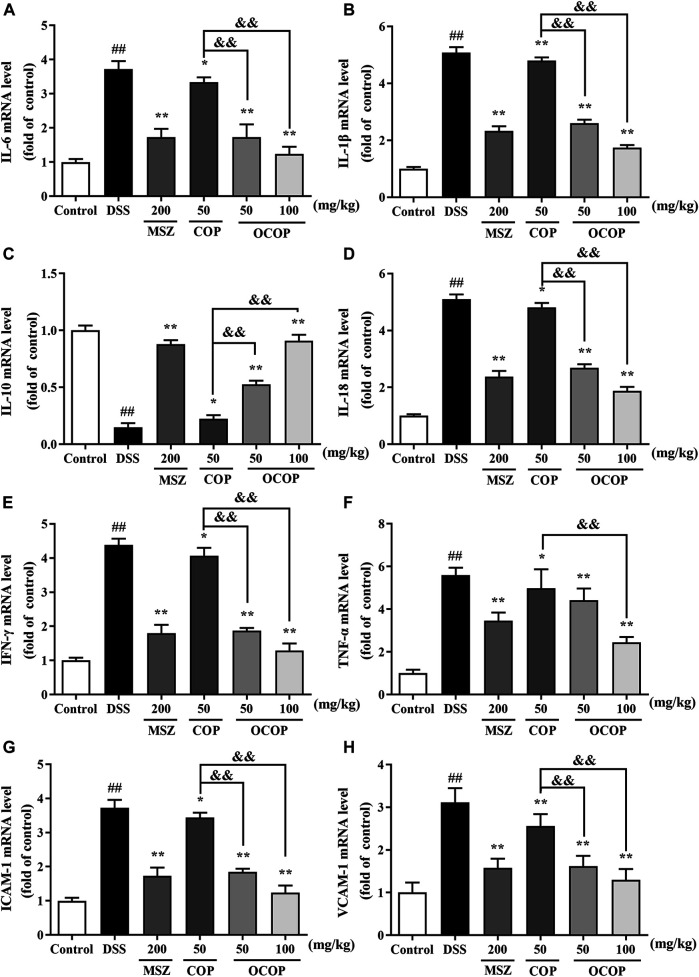
The mRNA expression levels of *IL-6* **(A)** *IL-1β* **(B)** *, IL-10* **(C)** *, IL-18* **(D)** *, IFN-γ* **(E)** *, TNF-α* **(F)** *, ICAM-1* **(G)** *, VCAM-1* **(H)** in mice as determined by qRT-PCR. Data are expressed as the means ± SD (n = 10). ^#^ *p* < 0.05, ^##^ *p* < 0.01 vs. control group; *p < 0.05, **p < 0.01 vs. DSS group; ^&^ *p* < 0.05, ^&&^ *p* < 0.01 vs. COP group.

As depicted in Figure 5, the mRNA expression of *IL-1β, IL-18, IL-6, TNF-α, IFN-γ, ICAM-1,* and *VCAM-1* was markedly (all *p* < 0.01) up-regulated in DSS-induced colitis group, while the *IL-10* expression was significantly (*p* < 0.01) down-regulated. However, OCOP, COP and MSZ treatment notably (*p* < 0.01) down-regulated the elevated expression levels of *IL-1β, IL-18, IL-6, TNF-α, IFN-γ, ICAM-1,* and *VCAM-1* induced by DSS. In addition, OCOP and MSZ remarkably (*p* < 0.05) promoted the expression of *IL-10.* Furthermore, OCOP (50 mg/kg) exhibited a more significant inhibitory effect on the expression of *IL-1β, IL-18, IL-6, TNF-α, IFN-γ, ICAM-1,* and *VCAM-1* than COP of the same dosage. The above results revealed that the anti-colitis effect of OCOP might be related to its modulation on the mRNA expression of immune-inflammation mediators.

#### Effect of 8-Oxocoptisne on the Expression of NLRP3 and p65 in the Colons

**FIGURE 6:**
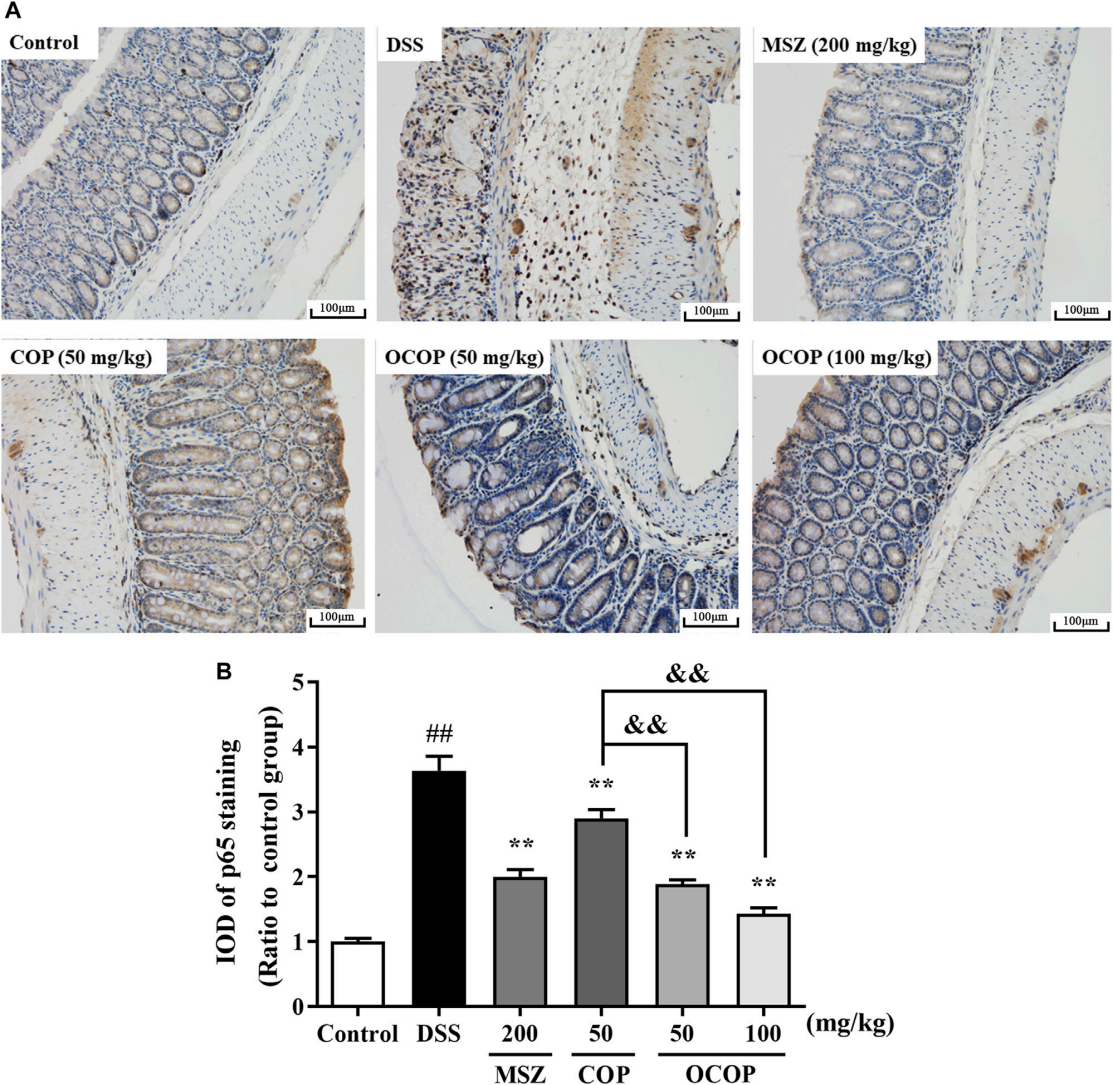
**(A)** Representative photographs of p65 immunohistochemical staining (magnification ×200) **(B)** Integrated optical density of p65. Data are expressed as the means ± SD (n = 3). ^*#*^ *p* < 0.05, ^##^ *p* < 0.01 vs. control group; *p < 0.05, **p < 0.01 vs. DSS group; ^&^ *p* < 0.05, ^&&^ *p* < 0.01 vs. COP group.

**FIGURE 7:**
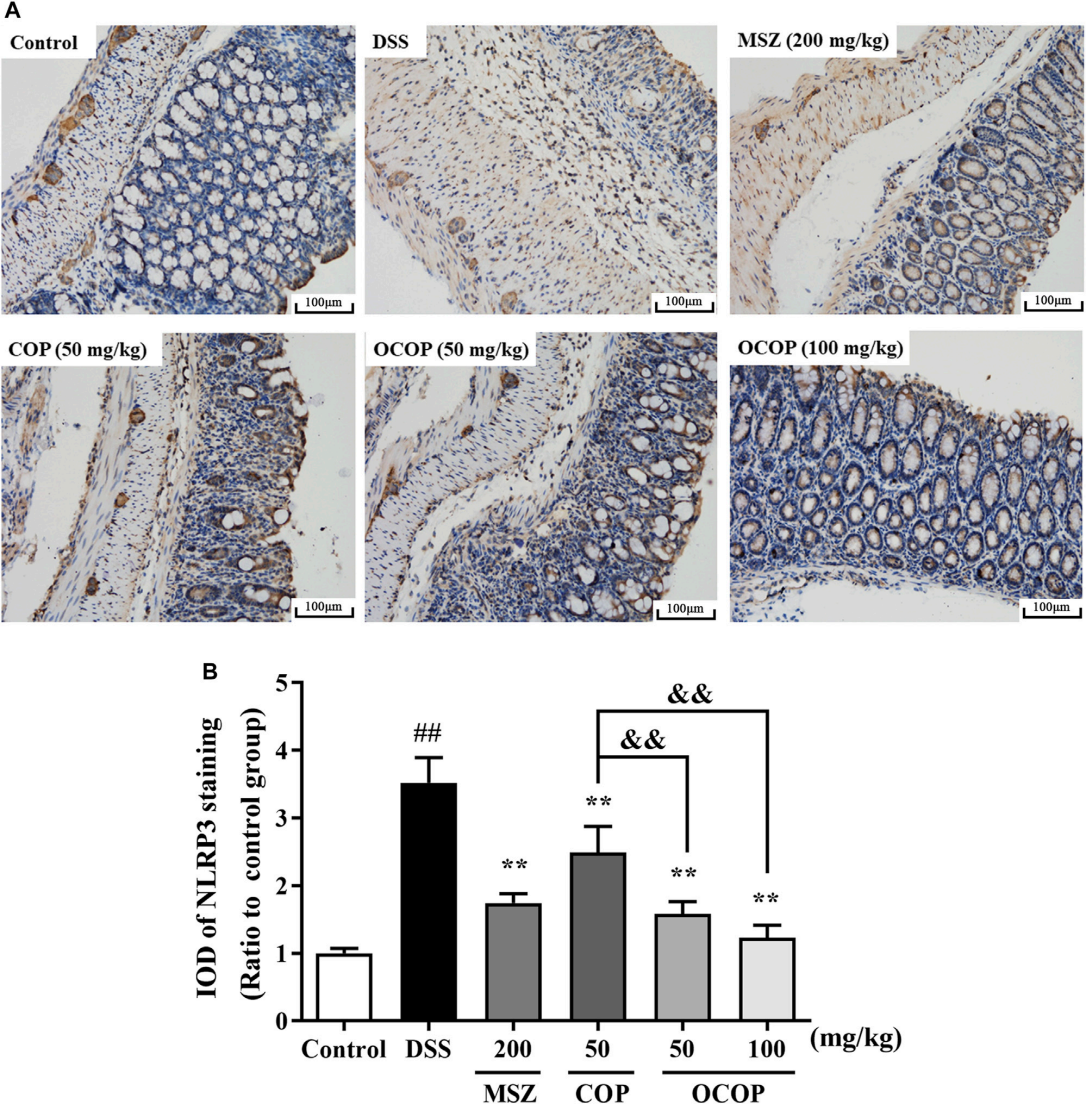
**(A)** Representative photographs of p65 immunohistochemical staining (magnification ×200) **(B)** Integrated optical density of p65. Data are expressed as the means ± SD (n = 3). ^*#*^ *p* < 0.05, ^##^ *p* < 0.01 vs. control group; *p < 0.05, **p < 0.01 vs. DSS group; ^&^ *p* < 0.05, ^&&^ *p* < 0.01 vs. COP group.

To further investigate the protective mechanism of OCOP against DSS-induced acute colitis, immunohistochemical evaluation of NLRP3 and p65 proteins was carried out. The result shown in Figure 6 indicated p65 subunit expression in the control group was relatively low. Compared to the control group, higher expression level was observed in the DSS model group. However, treatment with OCOP, COP and MSZ substantially (*p* < 0.01) reduced p65 staining in the colonic tissues. As illustrated in Figure 7, when compared to the control group, NLRP3 inflammasome was significantly (*p* < 0.01) elevated in DSS-induced mice. By contrast, administration with OCOP, COP and MSZ effectively (*p* < 0.01) attenuated the NLRP3 staining in colonic tissues.

#### Effect of 8-Oxocoptisne on Protein Expression of NF-κB Pathway and NLRP3 Inflammasome in DSS-Induced Colitis

**FIGURE 8:**
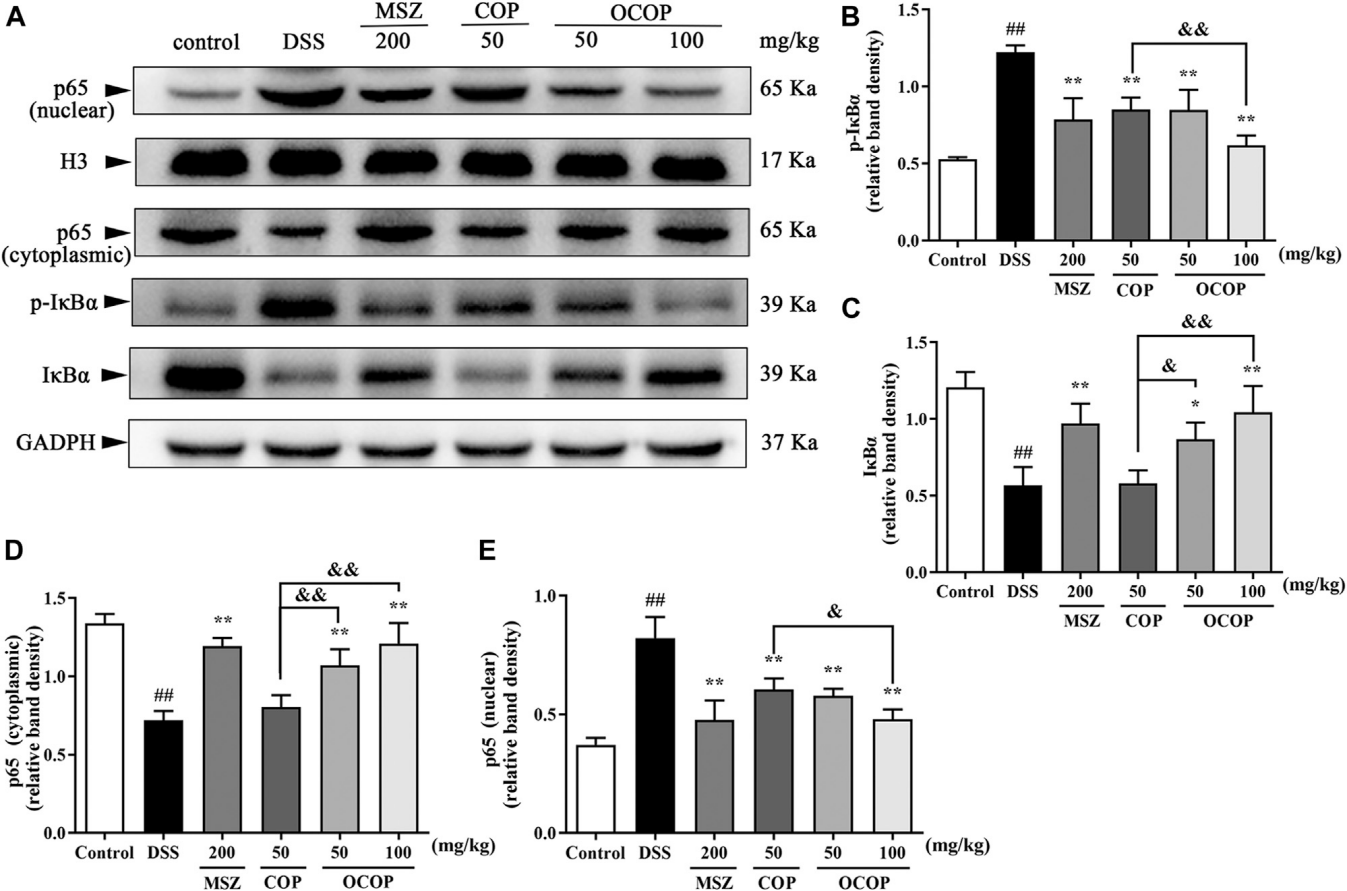
Effects of OCOP and COP on the NF-κB pathway in DSS-induced mice **(A)** Representative Western blotting images of pIκBα, IκBα, cytoplasmic p65 and nuclear p65 **(B–E)** The protein expression levels of pIκBα, IκBα, cytoplasmic p65 and nuclear p65 in the NF-κB pathway. Data are expressed as the means ± SD (n = 3). ^#^ *p* < 0.05, ^##^ *p* < 0.01 vs. control group; *p < 0.05, **p < 0.01 vs. DSS group; ^&^ *p* < 0.05, ^&&^ *p* < 0.01 vs. COP group.

**FIGURE 9:**
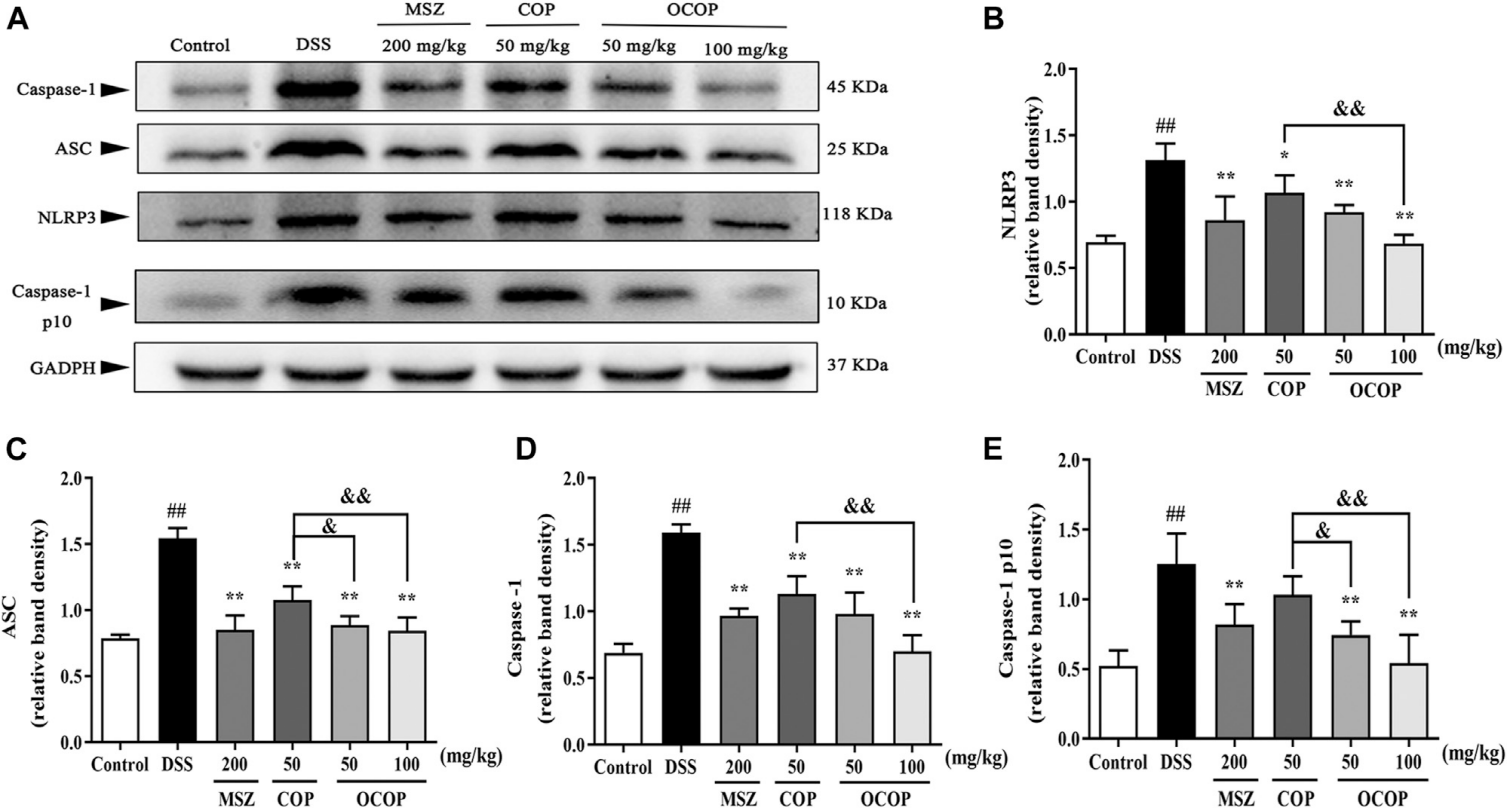
Effects of OCOP and COP on the NLRP3 inflammasome in DSS-induced mice **(A)** Representative Western blotting images of NLRP3, ASC, Caspase-1and Caspase-1 p10 **(B–E)** The protein expression levels of NLRP3, ASC, Caspase-1 and Caspase-1 p10 in the NLRP3 inflammasome. Data are expressed as the means ± SD (n = 3). ^#^ *p* < 0.05, ^##^ *p* < 0.01 vs. control group; *p < 0.05, **p < 0.01 vs. DSS group; ^&^ *p* < 0.05, ^&&^ *p* < 0.01 vs. COP group.

Long-term administration with DSS contributed to massive inflammation cells infiltration and a large amount of inflammatory cytokines secretion, leading to the occurrence of serious inflammation response. Therefore, to further explore whether the alleviative effect of OCOP and COP was associated with inhibition of NLRP3 inflammasome and NF-κB pathway in DSS-induced colitis, relevant signal molecules were examined. As illustrated in Figures 8, 9, the nuclear translocation of p65 was noticeably elevated (*p* < 0.01) when UC occurred, while treatment with OCOP, COP, and MSZ significantly (*p* < 0.05) inhibited p65 translocation from cytoplasm to nucleus in contrast to the model group.

Meanwhile, when compared to the control group, DSS exposure markedly (*p* < 0.01) enhanced the phosphorylation and degradation of IκB-α. Instead, as compared with the model group, treatment with OCOP, COP, and MSZ significantly (*p* < 0.05) suppressed the phosphorylation and degradation of IκB-α. Meanwhile, higher expression levels of NLRP3, ASC, Caspase-1 p10, and Caspase-1 were observed in DSS-induced mice. In contrast, the elevated expression levels of NLRP3, ASC, Caspase-1 p10, and Caspase-1 were markedly (*p* < 0.05) reduced by OCOP, COP, and MSZ, respectively.

#### Docking of Coptisine and 8-Oxocoptisne at the Potential Protein Active Site

**FIGURE 10:**
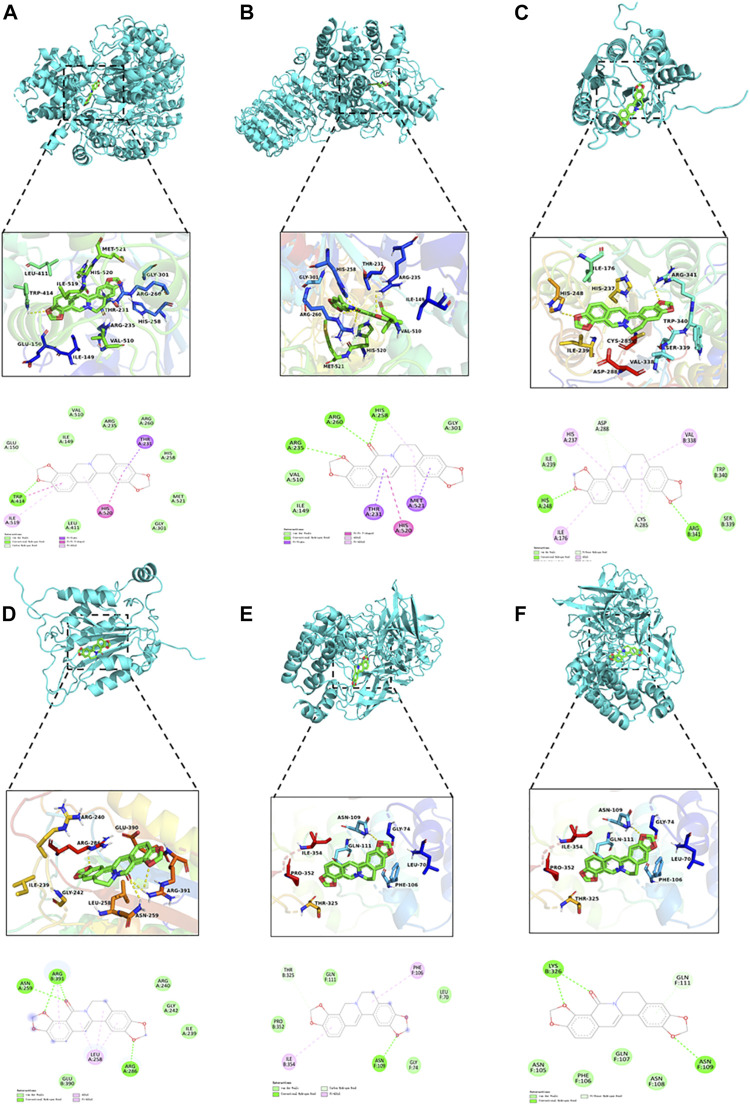
Molecular docking simulation of COP into the active site of NLRP3 **(A)**. OCOP in the active site of NLRP3 **(B)**. COP binding with Caspase-1 **(C)**. OCOP binding with Caspase-1 **(D)**. COP binding with NF-κB **(E)**. OCOP binding with NF-κB **(F)**.

As illustrated in Table 4 and Figure 10, the docking results showed that OCOP could combine with the active site of Caspase-1, NLRP3 and NF-κB with the binding energy of −8.2, −8.2 and −7.2 kcal/mol, respectively. Meanwhile, the docking interaction energy between COP and Caspase-1, NLRP3 and NF-κB was −6.7, −7.3 and −6.6 kcal/mol, respectively. COP interacted with ARG341, HIS248 of Caspase-1, TRP414 of NLRP3, ASN109 of NF-κB through hydrogen bonds. OCOP also formed hydrogen bonds with the amino acid ARG391, ASN259, ARG286 of Caspase-1, ARG260, ARG260, HIS258, ARG235 of NLRP3, and LYS326, ASN109 of NF-κB. These results suggested that OCOP and COP exhibited significant docking affinity to the binding sites of Caspase-1, NLRP3 and NF-κB.

**TABLE 4:** Hydrophobic interaction and docked amino acid residues of target proteins with OCOP and COP.

No.	Ligand	Target protein (PDB ID)	Binding energy	H-bond	Ligand atoms	Amino acid residue	H-bond length (Å)
1	OCOP	Caspase-1 (1RWK)	−8.2	4	C-3O	ARG391	3.1
					C-5O	ARG391	2.2
					C-5O	ASN259	2.8
					C-1O	ARG286	2.0
2	OCOP	NLRP3 (6NPY)	−8.2	4	C-5O	ARG260	3.1
					C-5O	ARG260	3.0
					C-5O	HIS258	2.4
					C-3O	ARG235	3.0
3	OCOP	NF-κB (1NFI)	−7.2	3	C-3O	LYS326	3.0
					C-5O	LYS326	2.6
					C-1O	ASN109	2.4
4	COP	Caspase-1 (1RWK)	−7.3	2	C-1O	ARG341	2.7
					C-4O	HIS248	2.4
5	COP	NLRP3 (6NPY)	−6.7	1	C-1O	TRP414	2.8
6	COP	NF-κB (1NFI)	−6.6	1	C-1O	ASN109	2.2

## Discussion

Rhizome Coptidis, a well-known herbal medicine with multiple applications including treatment for colitis in clinic, has attracted wide attention of scholars in China and elsewhere (Lee et al., 2014; Wu et al., 2019). Unsurprisingly, as one of the characteristic active principles of Rhizome Coptidis, COP could also exhibit similar beneficial effects on those diseases. However, low bioavailability but prominent pharmacological activities of COP has become a conundrum still not solved (He et al., 2016a; He et al., 2016b).

Since gut microflora-mediated conversion plays a crucial role in reducing the toxicities of some drugs or modulating their metabolism, especially those with poor bioavailability, gut metabolism was considered to be one of the main factors contributing to the pronounced pharmacological effects of drugs. It acts as a reservoir of genes encoding a board diversity of enzymes (reductase, oxidase, esterase, etc.), initiating a range of reactions *in vivo* such as oxidation, reduction, intramolecular cyclization, rearrangement, condensation, esterification, ester hydrolysis, isomerization, which are beneficial for both bacteria and host (Xu et al., 2017).

Among these metabolic reactions, oxidation is the common modification reaction. Yoo et al. have reported the oxidative metabolism of lovastatin was found on a potential gut microbiota-mediated drug-drug interaction, and it may be the material basis accounting for the excellent anti-colon cancer activity (Yoo et al., 2014). Shu et al. have reported levamisole can be oxidated into several thiazole ring-opened metabolites via gut microbiota, and then exerted the promising anti-tumor activity (Shu et al., 1991). In general, the oxidase enzymes in gut microbiota can convert prodrug into oxidative metabolites, which could be a principal element of these drugs to exhibit stronger therapeutic effects.

For the first time, the present study showed that the gut microbiota could be reduced in pseudo germ-free animal model, which was widely adopted to evaluate the relationship between microbiota xenobiotic metabolism and host (Kang et al., 2014). As expected, the process that COP was converted to OCOP was prominently inhibited by pretreatment with antibiotics. And combined with the results of molecular structure, NMR spectroscopy and HPLC-ESI-MS, gut microbiota transformed COP to a novel metabolite, OCOP, supposedly via oxidation reaction.

It is believed that, when COP was transformed into its oxidized metabolite OCOP, the lipophilicity would increase with the structure converted into more active lactam ring, which was beneficial to be absorbed through biofilm and improve its biological activity. Not unexpectedly, according to our preliminary experiment, COP and OCOP all exhibited prominent anti-colitis activity. However, comparative investigation on the anti-colitis effect of COP and OCOP is rare.

In this study, we adopted the colitis model induced by DSS, a well-characterized experimental colitis model for UC research (El-Shahat and Erfan, 2015; Zhao et al., 2015). Its various clinical manifestations closely resemble the clinical symptoms of human UC and are often regarded as the important indicators for evaluating the severity of the disease (Roger et al., 2011; Bardasi et al., 2020). The dramatic body weight loss, diarrhea, shorter colon length, rectal blood and higher DAI score were observed in colitis model mice, which indicated colitis mice model was established successfully. However, these deteriorations were all significantly ameliorated by treatment with OCOP, the effect of which was similar to MSZ with much smaller dosage, and superior to COP. Histopathological evaluation indicated that severe cell infiltration and disrupted epithelial cell layers could be observed in DSS-induced colitis. Nevertheless, pretreatment with OCOP notably ameliorated the inflammation and tissue damage as evident by the improved histological scores and decreased MPO activity, a biochemical marker reflecting the accumulation of neutrophils granulocyte into the inflamed intestinal tissue. These results suggested OCOP could effectively protect mice from DSS-induced colitis.

Inflammatory response, the main characteristic of gut barrier damage, plays a substantial role in the pathogenic process and management of UC (Xu et al., 2020). Increased levels of pro-inflammatory mediators and insufficient levels of anti-inflammatory cytokines are responsible for the development of inflammation in patients with UC (Schardey et al., 2019). The imbalance between pro-inflammatory and anti-inflammatory factors promotes the inflammation of mucosa and accelerates the incidence of UC (Yuan et al., 2019). Therefore, modulating favorably the imbalance may provide a vital potential strategy for alleviating the symptoms of UC.

To clarify the potential anti-inflammatory mechanism of OCOP, in the current study, relevant pro-inflammatory mediators, anti-inflammatory cytokines, and adhesion molecules in the colorectal tissues were determined. IL-18 is considered important pathogenic factor through reducing the number of goblet cells at the later stages of UC (Arend et al., 2008). In addition, IL-18 is also a pleiotropic cytokine contributing to production of interferon (IFN)-γ, which acts as an essential role in the aspect of macrophage activation (Takagawa et al., 2005). TNF-α, a potent pro-inflammation mediator, is secreted by macrophages and monocytes. Excessive TNF-α could lead to epithelial barrier damage, which is highly associated with the occurrence of UC and meanwhile triggers the generation of IL-6 and IL-1β to further deteriorate inflammatory process (Orlikova et al., 2013; Ahn and Kim, 2018). Besides, adhesion molecules are responsible for the severe intestinal damage and inflammatory cells infiltration. Studies have reported that the elevated levels of adhesion molecules (*ICAM-1* and *VCAM-1*) were founded in the DSS-induced colitis mice (Gulubova et al., 2007). In contrast, as a typical anti-inflammatory mediator, IL-10 can prevent exuberant immune response to pathogens and limit the excretion of several pro-inflammatory cytokines including IL-1β, IL-6, and TNF-α (Gabrysova et al., 2009). Simon et al. have built IL-10 deficiency mice to indicate that the occurrence of chronic intestinal inflammation was intimately related to IL-10 deficiency (Simon et al., 2016). TGF-β, a regulatory cytokine secreted both by intestinal epithelial cells and T cells, plays a vital role in regulating immunological homeostasis and inflammatory responses. The reduced TGF-β activity is responsible for the development of autoimmune disorders in several pathologic conditions including UC (Ihara et al., 2017; Zhu et al., 2019). In this study, significant up-regulated expression of above pro-inflammatory cytokines, adhesion molecules and TGF-β were observed in colitis model mice. On the contrary, pretreatment with OCOP dramatically reduced these elevated indicators. Furthermore, OCOP treatment markedly increased the depressed expression of IL-10 induced by DSS. This seemingly paradoxical increase in the anti-inflammatory TGF-β in DSS-induced colitis may be due to overexpression of SMAD7, which negatively regulated TGF-β signaling by competing for SMAD3’s binding site on the TGF-β receptor, thereby blocking SMAD3 activation (Feagins, 2010; Alliger et al., 2020). Therefore, shifting to maintain the equilibrium between these indicators may be important for OCOP to alleviate the inflammatory response.

Accumulating evidence has suggested that inflammatory mediators could activate NF-κB pathway and NLRP3 inflammasome (Shi et al., 2014a; Zhang et al., 2019). Those pathways, in turn, could also regulate the transcription of various inflammatory cytokines (Shi et al., 2014a). NF-κB signaling pathway is a classical pathway involved in modulation of inflammatory responses and immunity (Shi et al., 2014b). In an activated status, heterodimer comprising of NF-κB1/p50 and RelA/p65 is commonly sequestered in the cytoplasm through combining with its inhibitor protein IκB. And phosphorylation and degradation of IκB will trigger the dislocation of IκB and NF-κB when NF-κB is activated (Schuliga, 2015). Meanwhile, the liberated NF-κB dimers are translocated from cytoplasm to nucleus where NF-κB-related inflammatory mediators will be activated (Stephenson et al., 2000). In our study, results indicated that DSS could activate the nuclear translocation of p65 and facilitate phosphorylation and degradation of IκB. However, treatment with OCOP significantly reversed these changes. Therefore, OCOP might inhibit experimental colitis induced by DSS, at least in part, via suppressing the NF-κB pathway.

Apart from that, NF-κB related signaling pathway has been indicated to regulate NLRP3 expression (Shaker et al., 2014). And numerous studies have disclosed that the activation of NLRP3 inflammasome has been defined as a critical element of the pathogenic process of UC (Shen et al., 2019). NLRP3 inflammasome complex, an assembly constituted of NLRP3 itself, the adaptor ASC and the effector Caspase-1, could initiate inflammatory responses. It can be activated by a range of exogenous dangers, bacterium and virus, leading to the cleavage of ASC and activation of pro-Caspase-1 protein. Subsequently, the Caspase-1 protein is activated with vast cytokine IL-18 and IL-1β secretion, resulting in the inflammatory responses (Mogg, 2011; Platnich and Muruve, 2019). Perera et al. have reported MCC950, an inhibitor of NLRP3 inflammasome, could effectively attenuate colitis in DSS-induced mice (Perera et al., 2018). Therefore, suppressing the NLRP3 pathway and the subsequent cytokine expression might be beneficial for improving the symptoms of ulcerative colitis and preventing its recurrence. Remarkably, our data indicated pretreatment with OCOP significantly reduced the expression levels of NLRP3, ASC, and Caspase-1 to regulate NLRP3 inflammasome.

**FIGURE 11:**
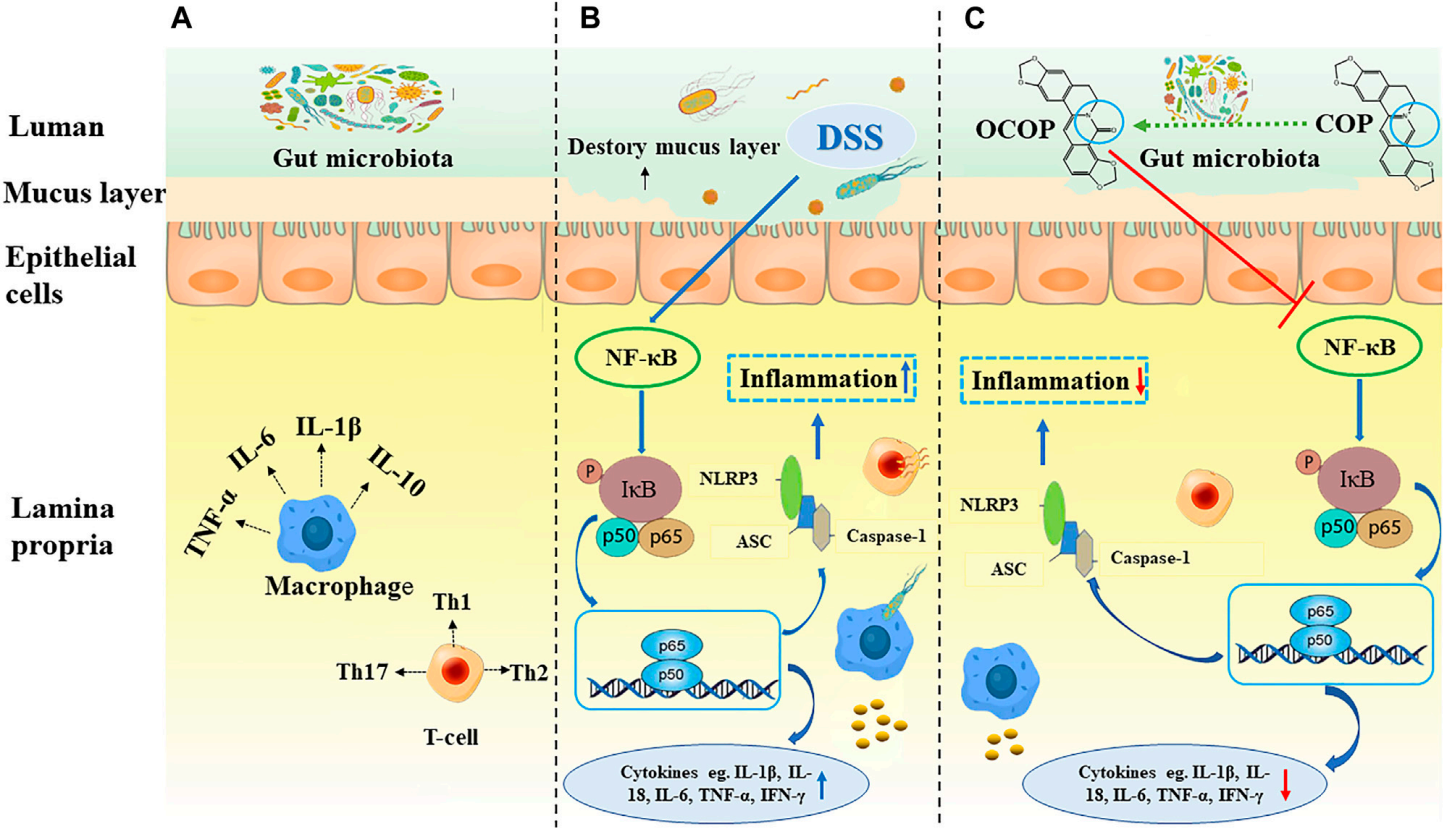
Summary scheme of the mechanisms underlying the inhibitory effect of OCOP **(A)** Normal intestinal tract **(B)** DSS-induced colitis **(C)** OCOP-pretreated colitis. OCOP, a novel gut microflora metabolite of COP, significantly blocked NF-κB pathway and NLRP3 inflammasome through down-regulating the protein expression of ASC, NLRP3 and Caspase-1, suppressing the phosphorylation of IκBα and the migration of NF-κB p65 from cytoplasm to nucleus.

It is interesting to note that, in our results, OCOP exerted superior effect to COP in the above aspects. And combined with the results of molecular docking, the protective effect on DSS-induced experimental colitis might be related to regulation of the NF-κB pathway and NLRP3 inflammasome (Figure 11).

This study was the pioneering effort to discover a novel metabolite of intestinal microflora of COP and report its ameliorative effect of OCOP against DSS-induced experimental acute colitis. The work provided empirical experimental foundation to support the traditional application of *Coptis Chinemsis* Franch in the treatment of dysentery. The results provided further insight into the role of microbial metabolism in the transformation and biological manifestation of natural products like COP, and its oxidative metabolite OCOP stands the chance to be further developed into an alternative lead compound for the treatment of colitis. However, more efforts are merited to investigate the underlying mechanism and potential benefit of OCOP in more detail, even in other different experimental models.

In conclusion, OCOP, a novel metabolite of intestinal microflora of COP, notably ameliorated clinical manifestations, colonic injury, and inflammatory response against colitis, which was associated with the suppression of the NF-κB pathway and NLRP3 inflammasome as well as subsequent regulation of the imbalance between pro-inflammatory and anti-inflammatory mediators. Noteworthily, OCOP was observed to exhibit similar therapeutic effect to MSZ with much smaller dosage, and was superior to COP, which indicated that OCOP might have greater potential to be further exploited as a promising therapeutic candidate in the treatment of UC.

## Data Availability

The raw data supporting the conclusions of this article will be made available by the authors, without undue reservation.

## Abbreviations

COP, coptisine; DAI, disease activity index; DSS, dextran sulfate sodium; ELISA, enzyme-linked immunosorbent assay; H&E, hematoxylin and eosin; IBD, inflammatory bowel disease; IFN-γ, interferon-γ; IL-10, interleukin-10; IL-18, interleukin-18; IL-β, interleukin-1β; IL-6, interleukin-6; ICAM-1, intercellular cell adhesion molecule 1; MPO, myeloperoxidase; MSZ, mesalazine; OCOP, 8-oxocoptisne; TNF-α, tumor necrosis factor-α; TGF-β, Transforming growth factor-β; UC, ulcerative colitis; VCAM-1, vascular cell adhesion molecule 1.
