# Supplementary material for: Gut Microbiota-Mediated Transformation of Coptisine Into a Novel Metabolite 8-Oxocoptisine: Insight Into Its Superior Anti-Colitis Effect
Source: Front Pharmacol. 2021 Mar 30;12:639020. doi: 10.3389/fphar.2021.639020 (PMC8042337; doi:10.3389/fphar.2021.639020)
Supplement: Supplementary file 1 [file datasheet1.docx]

**Tables**

**Table 1** Criteria for scoring disease activity index (DAI).

| Score | Body weight (compared to the original body weight) | Stool bleeding | Stool consistency |
| --- | --- | --- | --- |
| 0 | No change | None | Normal |
| 1 | ≤ 5% |  | Soft but still formed |
| 2 | ≤ 6~10% | Blood traced in stool visible | Very soft |
| 3 | ≤ 11~20% |  | Half diarrhea |
| 4 | ≥ 20% | Totally rectal bleeding | Diarrhea |

**Table 2** Histological grading criteria.

| Score | Epithelial loss | Crypt damage | Reduction in the number of goblets cells | Inflammatory cell infiltration |
| --- | --- | --- | --- | --- |
| 0 | no loss | no damage | none | none |
| 1 | 0 ~ 5% | 5 ~ 10% damage | mild | mild |
| 2 | 5 ~ 10% | 10 ~ 20% damage | moderate | moderate |
| 3 | more than 10% loss | more than 20% damage | severe | severe |

**Table 3** Primers sequences

| Gene |  | Gene sequence (5’ to 3’) |
| --- | --- | --- |
| *GAPDH* | Forward | GCACAGTCAAGGCCGAGAATGG |
|  | Reverse | GGTGGCAGTGATGGCATGGAC |
| *ICAM-1* | Forward | AGTCGTCCGCTTCTACC |
|  | Reverse | CCAGCACCGTGAATGTGATCTCC |
| *VCAM-1* | Forward | TGTGCTGCTATTGGCTGTGACTC |
|  | Reverse | GCAGTTGACAGTGACAGGTCTCC |
| *TNF-α* | Forward | GCGACGTGGAACTGGCAGAAG |
|  | Reverse | CATCGGCTGGCACCACTAGTTG |
| *IL-1β* | Forward | GCACTACAGGCTCCGAGATGAAC |
|  | Reverse | AGGCTTGTGCTCTGCTTGTGAG |
| *IL-18* | Forward | TGCCATGTCAGAAGACTCTTGCG |
|  | Reverse | GGTCACAGCCAGTCCTCTTACTTC |
| *IL-10* | Forward | AGCTGGACAACATACTGCTAACCG |
|  | Reverse | CTTCACCTGCTCCACTGCCTTG |
| *IFN-γ* | Forward | TTACTGCCACGGCACAGTCATTG |
|  | Reverse | TCGCCTTGCTGTTGCTGAAGAAG |
| *IL-6* | Forward | TGAACAACGATGATGCACTTGCAG |
|  | Reverse | TAGCCACTCCTTCTGTGACTCCAG |

**Table 4** Hydrophobic interaction and docked amino acid residues of target proteins with OCOP and COP.

| No. | Ligand | Target protein (PDB ID) | Binding energy | H-bond | Ligand  atoms | Amino acid residue | H-bond length  (Å) |
| --- | --- | --- | --- | --- | --- | --- | --- |
| 1 | OCOP | Caspase-1 (1RWK) | -8.2 | 4 | C-3O | ARG391 | 3.1 |
|  |  |  |  |  | C-5O | ARG391 | 2.2 |
|  |  |  |  |  | C-5O | ASN259 | 2.8 |
|  |  |  |  |  | C-1O | ARG286 | 2.0 |
| 2 | OCOP | NLRP3 (6NPY) | -8.2 | 4 | C-5O | ARG260 | 3.1 |
|  |  |  |  |  | C-5O | ARG260 | 3.0 |
|  |  |  |  |  | C-5O | HIS258 | 2.4 |
|  |  |  |  |  | C-3O | ARG235 | 3.0 |
| 3 | OCOP | NF-κB (1NFI) | -7.2 | 3 | C-3O | LYS326 | 3.0 |
|  |  |  |  |  | C-5O | LYS326 | 2.6 |
|  |  |  |  |  | C-1O | ASN109 | 2.4 |
| 4 | COP | Caspase-1 (1RWK) | -7.3 | 2 | C-1O | ARG341 | 2.7 |
|  |  |  |  |  | C-4O | HIS248 | 2.4 |
| 5 | COP | NLRP3 (6NPY) | -6.7 | 1 | C-1O | TRP414 | 2.8 |
| 6 | COP | NF-κB (1NFI) | -6.6 | 1 | C-1O | ASN109 | 2.2 |
