# Supplementary material for: Gut Microbiota-Mediated Transformation of Coptisine Into a Novel Metabolite 8-Oxocoptisine: Insight Into Its Superior Anti-Colitis Effect
Source: Front Pharmacol. 2021 Mar 30;12:639020. doi: 10.3389/fphar.2021.639020 (PMC8042337; doi:10.3389/fphar.2021.639020)
Supplement: Supplementary file 2 [file datasheet2.docx]

Repeat1 Repeat2 Repeat3







**A**

GAPDH









**B**

ASC









**C**

NLRP3









**D**

Caspase-1









**E**

p65

(nuclear)









**F**

p65

(cytoplasmic)









**G**

IκBα







  **H**

p-IκBα









**I**

H_3_

**Supplementary Figure: Original images for A (GAPDH), B (ASC), C (NLRP3), D (Caspase-1), E [p65(nuclear)], F [ p65 (cytoplasmic)], G (IκBα), H (p-IκBα) and I (H3) blots in the manuscript.**

The band within the red frame is the target band. Control, DSS, MSZ (200 mg/kg), COP (50 mg/kg), OCOP (50 mg/kg), and OCOP (100 mg/kg)
