# Supplementary material for: Gut Microbiota-Mediated Transformation of Coptisine Into a Novel Metabolite 8-Oxocoptisine: Insight Into Its Superior Anti-Colitis Effect
Source: Front Pharmacol. 2021 Mar 30;12:639020. doi: 10.3389/fphar.2021.639020 (PMC8042337; doi:10.3389/fphar.2021.639020)
Supplement: Supplementary file 3 [file datasheet3.doc]

Repeat1 Repeat2 Repeat3


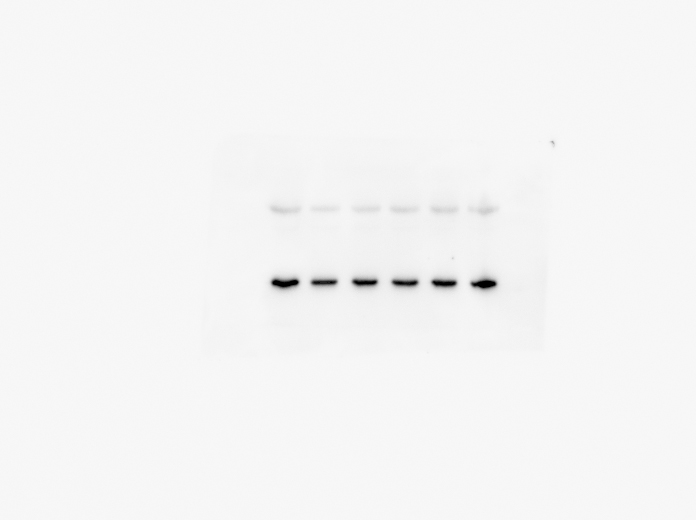

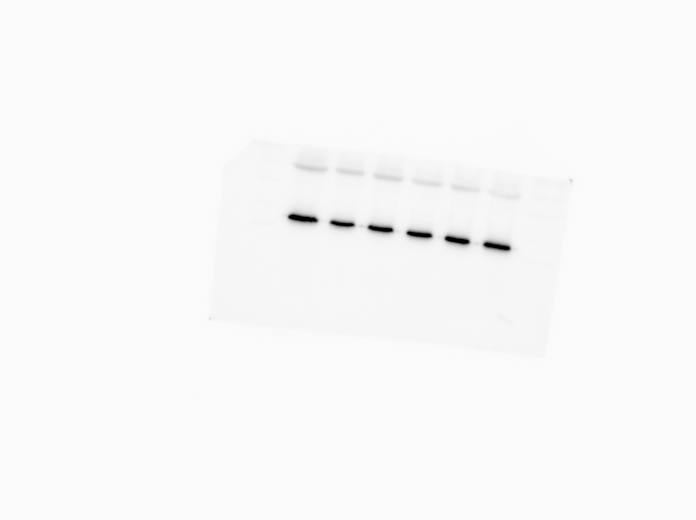

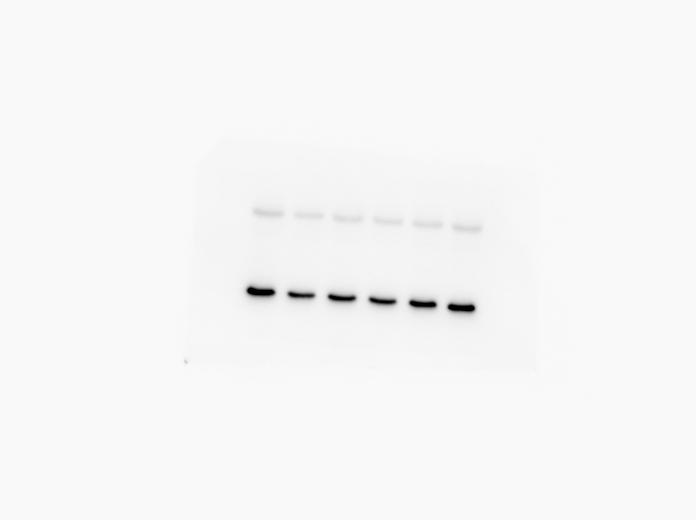
 **A**

Bcl-2


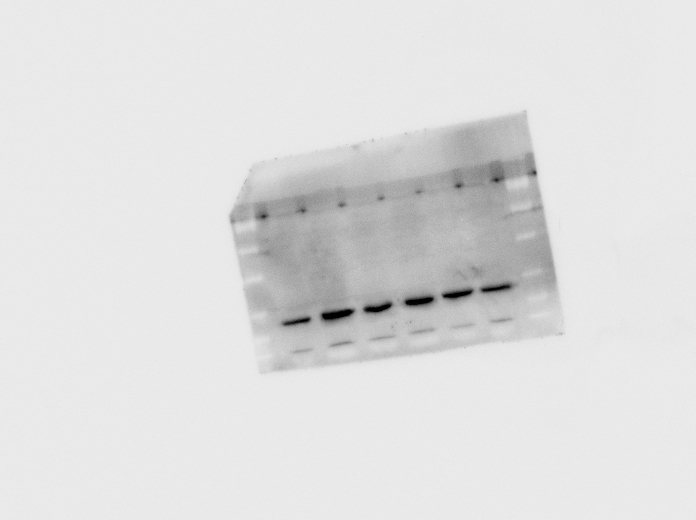

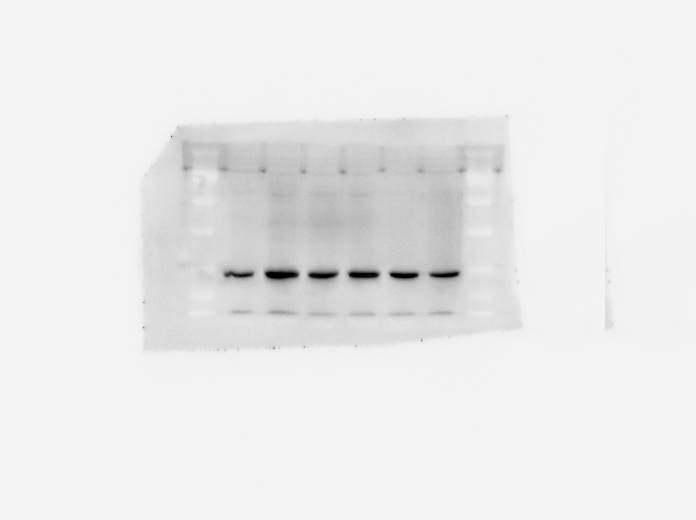

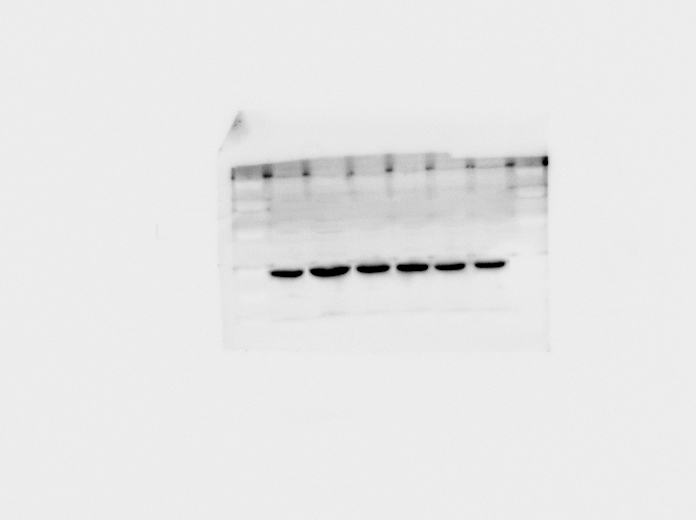
**B**

Bax


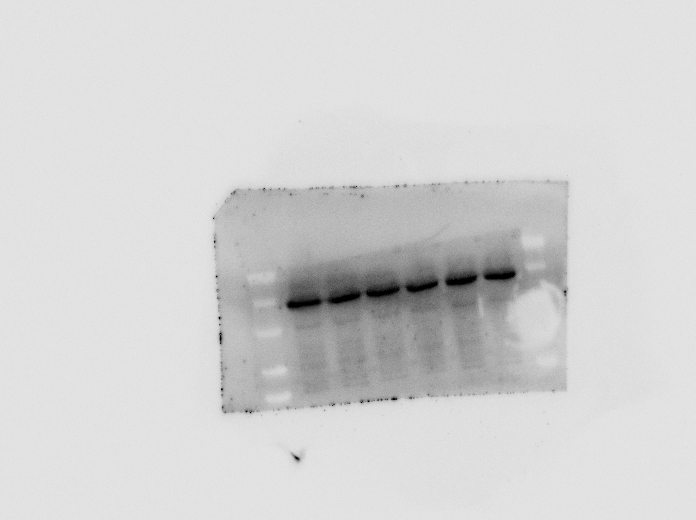

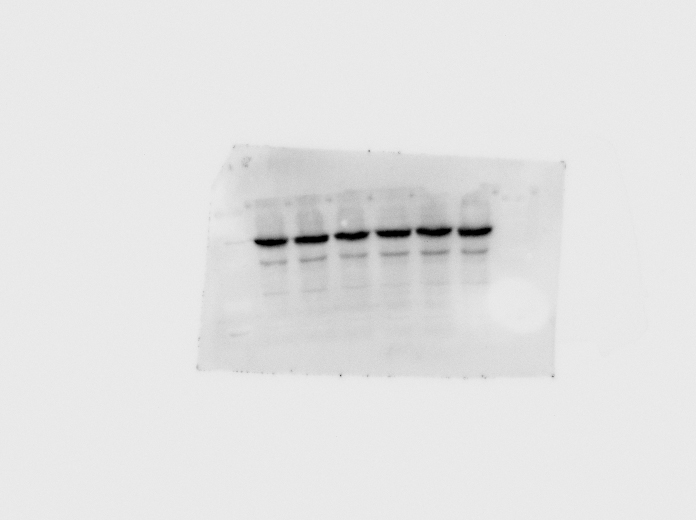

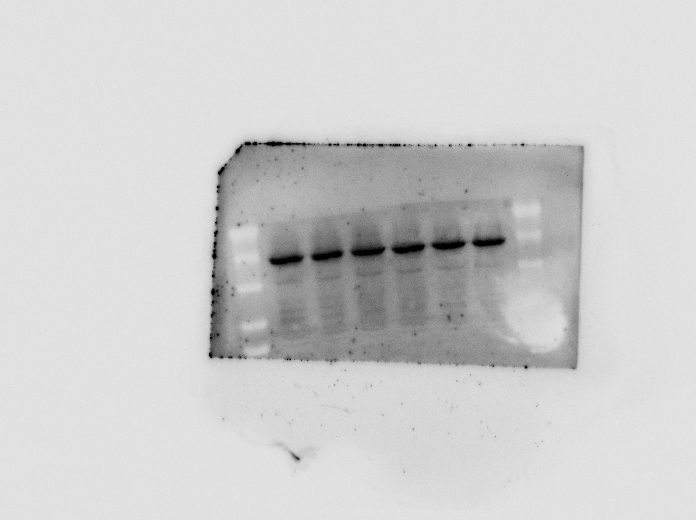
**C**

JNK


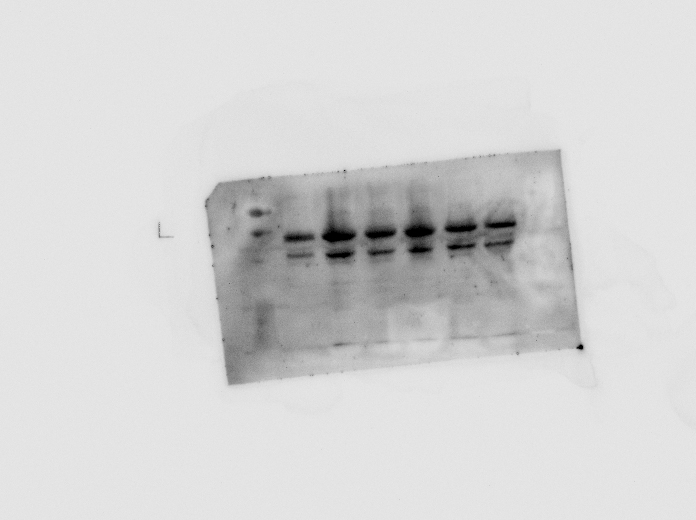

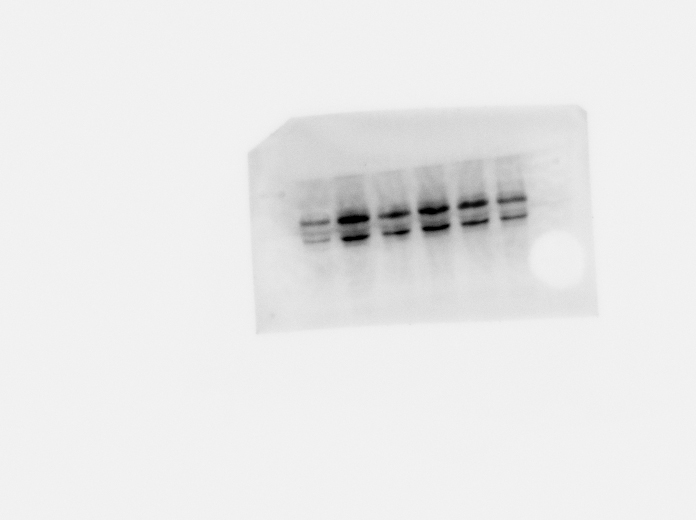

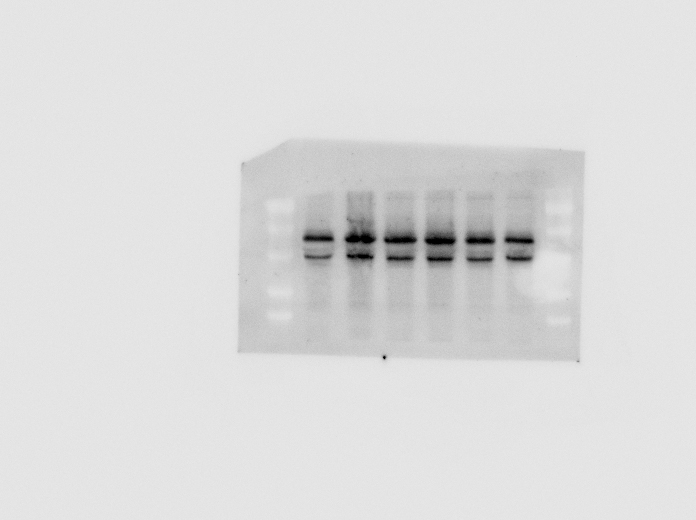
**D**

p-JNK

**E** Caspase-1 p10


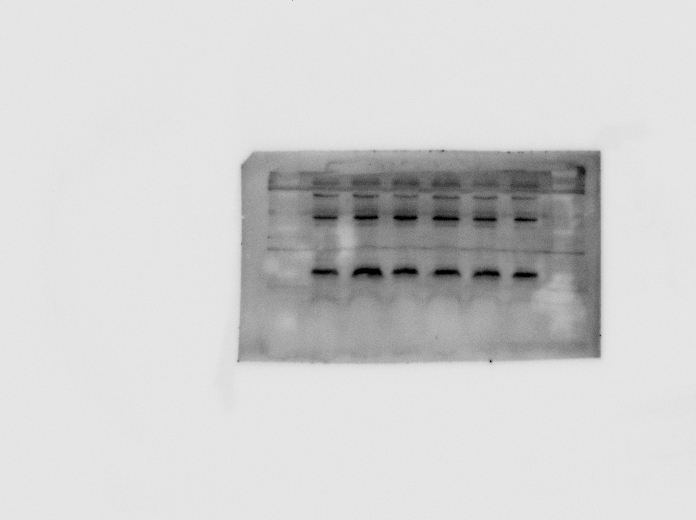

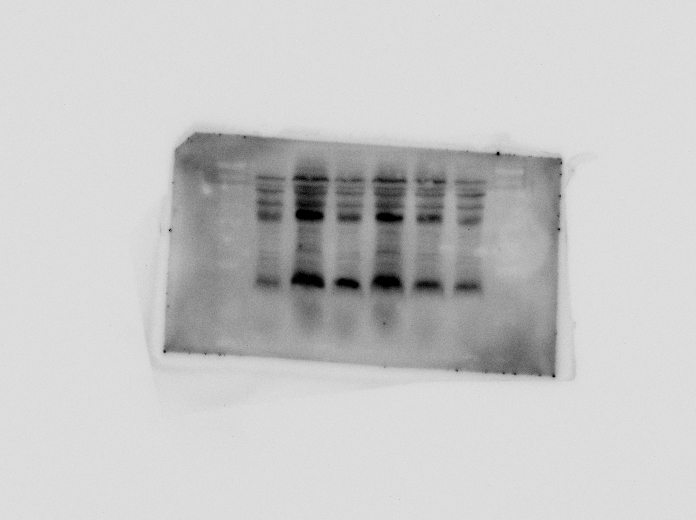

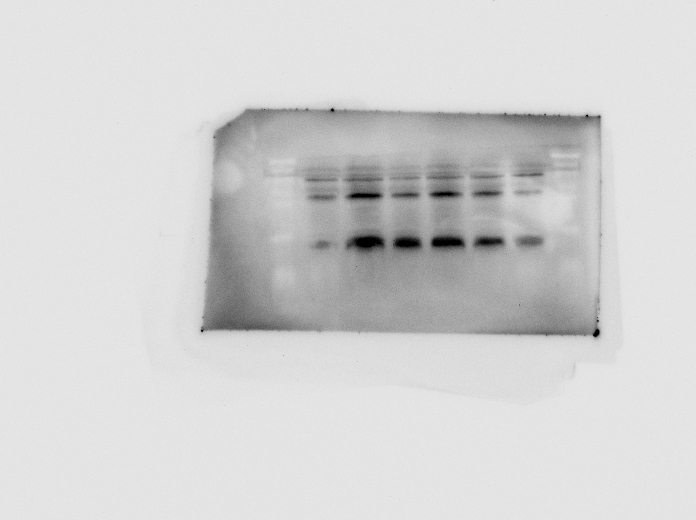


**Supplementary Figures: Original images for A (Bcl-2), B (Bax), C (JNK), D (p-JNK) and E (Caspase-1 p10) blots in the manuscript.**

The band within the red frame is the target band. Control, DSS, MSZ (200 mg/kg), COP (50 mg/kg), OCOP (50 mg/kg), OCOP (100 mg/kg).


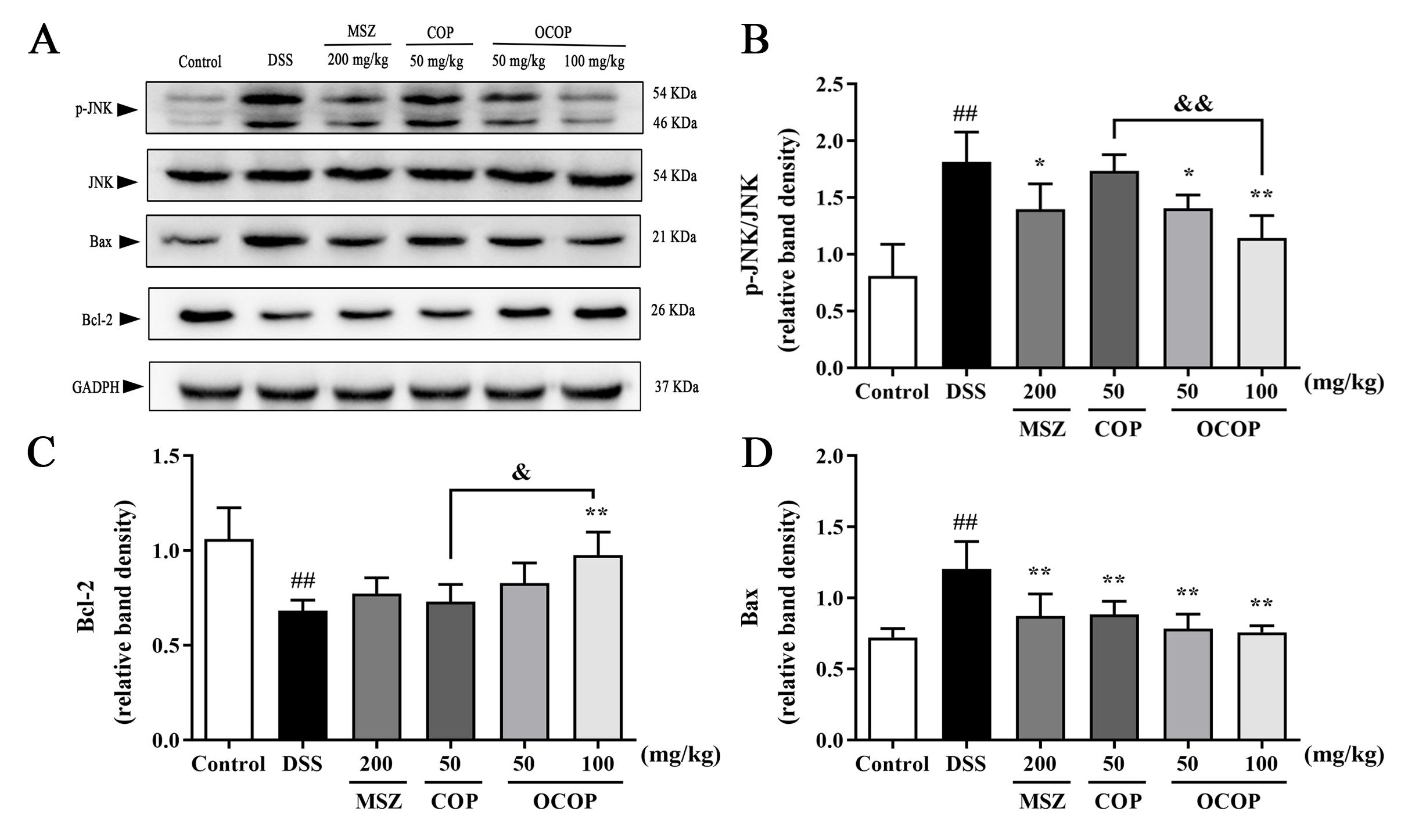


**Figure. 1**

**Fig. 1.** Effects of OCOP and COP on the apoptosis-related signaling pathway in DSS-induced mice. (A) Representative Western blotting images of p-JNK, JNK, Bax and Bcl-2. (B-D) The protein expression levels of p-JNK/JNK, Bcl-2 and Bax in the apoptosis-related signaling pathway. Data are expressed as the means ± SD (n = 3). ^#^*p* < 0.05, ^##^*p* < 0.01 vs. control group; **p* < 0.05, ***p* < 0.01 vs. DSS group; ^&^*p* < 0.05, ^&&^*p* < 0.01 vs. COP group.

**Result:** In our findings, the expression of Bax in the DSS group was markedly enhanced compared to the control group, while COP (50 mg/kg) and OCOP (50 mg/kg, 100 mg/kg) treatment effectively (P < 0.01) suppressed the increase of Bax expression. On the contrary, the expression of Bcl-2 was decreased in the DSS group, while OCOP (100 mg/kg) treatment counteracted the decrease of Bcl-2. In the meanwhile, we also analyzed the expression of JNK and p-JNK, which also play a vital role in the process of apoptosis. DSS significantly induced strong phosphorylation (activation) of JNK, however, the increased phosphorylation of JNK was suppressed by OCOP (50 mg/kg, 100 mg/kg).

**Discussion:** Recently, increasing evidences have demonstrated the increased inflammatory cytokines not only aggravated colitis but also promoted the apoptosis of intestinal epithelial cells, which involved in the pathogenesis of IBD (Roy et al., 2008; Kumar et al., 2015; Chu et al., 2021). c-Jun N-terminal kinase (JNK), the member of the MAPK family, has been reported to play a key role promoting apoptosis (Gao et al., 2019; Wu et al., 2020). In the intracellular machinery of apoptosis, Bcl-2 family proteins consists of members that inhibit apoptosis (Bcl-2) and promote apoptosis (Bax), which mainly control intrinsic pathway (Siddiqui et al., 2015; Adamkov, 2019). The increased number of apoptotic epithelial cells enhanced Bax but decreased Bcl-2 protein during active UC may result in the disruption of intestinal barrier, which contributed to abundant pathogenic microorganism infiltration (Yin et al., 2020). In line with previous reports, the elevated Bax and reduced Bcl-2 were observed in the DSS group. In contrast, OCOP treatment normalized the expression of Bax and Bcl-2. DSS significantly induced strong phosphorylation (activation) of JNK, and the increased phosphorylation of JNK was suppressed by OCOP (50 mg/kg, 100 mg/kg). Therefore, OCOP protected from the experimental colitis elicited by DSS, at least in part, through inhibiting epithelial apoptosis.

**References**

Adamkov, M. (2019). Logical complexity of Bcl-2 family proteins function in the intrinsic apoptosis. *Srpski Arhiv Za Celokupno Lekarstvo* 147(1-2)**,** 99-104. doi: 10.2298/sarh190124010a.

Chu, X., Wang, C., Wu, Z., Fan, L., Tao, C., Lin, J., et al. (2021). JNK/c-Jun-driven NLRP3 inflammasome activation in microglia contributed to retinal ganglion cells degeneration induced by indirect traumatic optic neuropathy. *Experimental eye research* 202**,** 108335-108335. doi: 10.1016/j.exer.2020.108335.

Gao, W.Y., Wang, C.H., Yu, L., Sheng, T.J., Wu, Z.L., Wang, X.Q., et al. (2019). Chlorogenic Acid Attenuates Dextran Sodium Sulfate-Induced Ulcerative Colitis in Mice through MAPK/ERK/JNK Pathway. *Biomed Research International* 2019**,** 13. doi: 10.1155/2019/6769789.

Kumar, A., Singh, U.K., Kini, S.G., Garg, V., Agrawal, S., Tomar, P.K., et al. (2015). JNK pathway signaling: a novel and smarter therapeutic targets for various biological diseases. *Future Medicinal Chemistry* 7(15)**,** 2065-2086. doi: 10.4155/fmc.15.132.

Roy, P.K., Rashid, F., Bragg, J., and Ibdah, J.A. (2008). Role of the JNK signal transduction pathway in inflammatory bowel disease. *World Journal of Gastroenterology* 4(2)**,** 200-202. doi: 10.3748/wjg.14.200.

Siddiqui, W.A., Ahad, A., and Ahsan, H. (2015). The mystery of BCL2 family: Bcl-2 proteins and apoptosis: an update. *Archives of Toxicology* 89(3)**,** 289-317. doi: 10.1007/s00204-014-1448-7.

Wu, Q.H., Wu, W.D., Jacevic, V., Franca, T.C.C., Wang, X., and Kuca, K. (2020). Selective inhibitors for JNK signalling: a potential targeted therapy in cancer. *Journal of Enzyme Inhibition and Medicinal Chemistry* 35(1)**,** 574-583. doi: 10.1080/14756366.2020.1720013.

Yin, S.J., Yang, H.F., Tao, Y., Wei, S.M., Li, L.H., Liu, M.J., et al. (2020). Artesunate ameliorates DSS-induced ulcerative colitis by protecting intestinal barrier and inhibiting inflammatory response. *Inflammation* 43(2)**,** 765-776. doi: 10.1007/s10753-019-01164-1.
